# A Novel Approach for the Synthesis of Peripherally Acting Dual Target Inhibitor of Cannabinoid-1 (CB1 Receptor) and Inducible Nitric Oxide Synthase (iNOS) (*S*-MRI-1867/Zevaquenabant)

**DOI:** 10.3390/molecules31030515

**Published:** 2026-02-02

**Authors:** Malliga R. Iyer

**Affiliations:** Section on Medicinal Chemistry, National Institute on Alcohol Abuse and Alcoholism, National Institutes of Health, 5625 Fishers Lane, Rockville, MD 20852, USA; malliga.iyer@nih.gov

**Keywords:** CB_1_R CB_2_R, ECS, iNOS, MASH, SFC, PSA, MPO

## Abstract

Zevaquenabant (*S*-MRI-1867) is a clinical-stage agent that is a peripherally restricted, potent antagonist of CB_1_R and an inhibitor of inducible nitric oxide synthase. A novel synthetic route to this highly selective active pharmaceutical agent is described in this paper. This route makes use of rationally installed chiral thio-substituted leaving group derived from a Bunte-salt reaction approach to yield diastereomeric compounds which are further processed to enantiopure compounds. The method will enable a rapid assembly of a variety of chiral sulfonyl amino compounds in this series.

## 1. Introduction

Physiological and pathological processes in the mammalian system are governed by a vast network of signaling pathways—one of which has significant contributions from the endocannabinoid system (ECS) [1,2,3]. The ECS encompasses a plethora of signaling baselines and endzones including GPCRs, enzymes, and ion channels [4]. The two best characterized receptors of the ECS include seven-trans membrane G-protein coupled receptor subtypes CB_1_R and CB_2_R [5]. The receptors bind endogenous lipid molecules called endocannabinoids (EC) found throughout the body along with exogenous ligands that could be manipulated to alter endocannabinoid-implicated processes [4,6,7].

Abundance of the CB_1_R in the brain, makes it responsible for the psychoactive effects of marijuana’s active component THC and exogenous synthetic agonists [8,9,10,11]. Synthetic cannabinoids exert behavioral effects on memory, appetite, and motor functions though their CB_1_R CNS target engagement. The past two decades of research shows that significant physiological processes can be modulated by the presence of CB_1_R in a number of peripheral tissues like lung, liver, and kidney [12,13,14,15]. The CB_2_R is mainly found in the cells of the immune system and involved in the immunomodulatory effects induced by cannabinoids [16]. Recent evidence supports that these receptors also exist in the CNS and may have a therapeutic role in modulation of neurodegenerative diseases [17,18].

An overactive CB_1_R system has contributions in the genesis of metabolic disorders like obesity, type 2 diabetes, dyslipidemia, and liver fibrosis [19]. CB_1_R blockade has shown beneficial effects in mitigating these conditions in animal models as well as in obese individuals with metabolic syndrome [20]. The anorexigenic effects mediated by CB_1_R blockade resulted in the approval of the first-in-class CB_1_R inverse agonist Rimonabant in the EU, although not by the US FDA. Subsequent suicidal ideation, and other neuropsychiatric liabilities, resulting from global CB_1_R blockade led to the withdrawal of this drug from the European market [21]. While this dealt a blow to the clinical development of the CB_1_R antagonist class of drugs already in the pipeline, the modulation of endocannabinoid functions through CB_1_R blockade has remained a viable therapeutic strategy for the treatment of obesity and its metabolic complications [7,22,23,24]. This concept has gained traction due to a continuously emerging body of evidence suggesting that the beneficial effects of CB_1_R blockade can be retained and the psychiatric side-effects can be negated by limiting CB_1_R blockade to the periphery [25]. Hence, novel strategies are being explored and are currently in clinical development [26] to sequester the CB_1_R blocking compounds away from the brain and restricted to the periphery. Within that paradigm, our approach was to develop compounds that by design would not only limit its blood–brain barrier (BBB) penetration but could also offer an additional benefit of targeting a secondary enzymatic/receptor signaling pathway [27,28,29]. The general strategy for designing non-brain penetrant compounds are well validated [30]. Subsequently, numerous small molecule peripheral agents acting as CB1 antagonists have been discovered [26,31,32].

MRI-1891 (monlunabant) is an example of a peripherally selective cannabinoid receptor 1 (CB1) inverse agonist being developed as a novel anti-obesity agent and for metabolic disorders such as diabetic kidney disease (DKD) discovered in our lab. Recent phase 2a trials conducted by Inversago/Novo Nordisk showed the efficacy of once-daily oral doses of 10 mg, 20 mg, and 50 mg monlunabant vs. placebo in adults with obesity and metabolic syndrome. All doses produced statistically significant and clinically meaningful weight loss compared to placebo, but the most effective and well-tolerated dose was 10 mg [33,34].

A promising therapeutic target involved in metabolic disorders, fibrosis, and its related pathologies is inducible nitric oxide synthase (iNOS), an enzyme that catalyzes the generation of reactive nitrogen species involved in inflammatory pathways. Increased iNOS activity contributes to diet-induced steatohepatitis, insulin resistance and obesity-associated inflammation and endoplasmic reticulum dysfunction [35,36]. Hepatic macrophages generating iNOS have been identified as the main fibrogenic cell population in experimental liver fibrosis [36]. In many pathophysiological processes, the role of iNOS mirrors CB1 overactivation [32]. Thus, a combined inhibition of iNOS and blockade of CB_1_R restricted to the periphery can be a promising single molecule, ‘two-pronged’ approach to treat fibrosis and its underlying conditions. This objective was realized with the disccovery of *S*-MRI-1867 (Zevaquenabant) a small-molecule drug designed as a third-generation cannabinoid receptor 1 (CB1) antagonist that is both peripherally selective and exhibits additional pharmacological activity as an inducible nitric oxide synthase (iNOS) inhibitor [27,28,37]. By targeting both CB1 receptors (outside the central nervous system) and iNOS, zevaquenabant aims to address diseases involving fibrosis and metabolic dysfunction.

**Figure 1:**
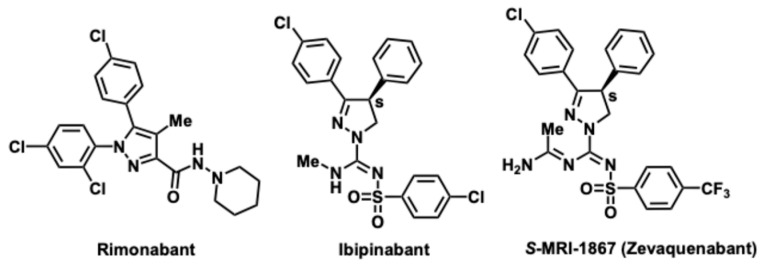
Centrally acting and peripherally restricted CB_1_R inverse agonists.

Replacement of the N-methyl group of ibipinabant with pendant acetamidine, a known pharmacophore in iNOS inhibitors [38], was found to be particularly attractive because it resulted in excellent CB_1_R affinity, favorable physicochemical parameters as well as peripheral restriction (Figure 1).

Numerous preclinical studies have now appeared in the literature that shows the superior efficacy of *S*-MRI-1867 as compared to drugs that engage only one of these targets [27,28,39].

A first-in-human Phase 1 clinical trial (NCT04531150) evaluated single and multiple ascending oral doses of *S*-MRI-1867 as INV-101 (zevaquenabant) in healthy adult volunteers for safety, tolerability, and pharmacokinetics. This trial has been completed with future development planned for treating lung and metabolic diseases.

The recent insights into the cryoEM structures of inactive state CB1 bound to peripheral antagonists along with previously revealed X-ray crystal structures of antagonist-bound human CB1R is expected to further enhance the rational design of next-generation CB1R antagonists [40,41].

As newer four-arm CB1 antagonists redefine the paradigm of peripheral CB1 blockade, novel synthetic pathways need to be enabled to realize extensive library generation and related structure function studies. Herein we selected the compound *S*-MRI-1867 as a prototype to showcase our new synthetic methodology and allow access to chiral eutomer of racemic MRI-1867 that belong to the ‘four-arm’ CB1 antagonist class of compounds.

## 2. Results and Discussion

### Chemistry

**Figure 2:**
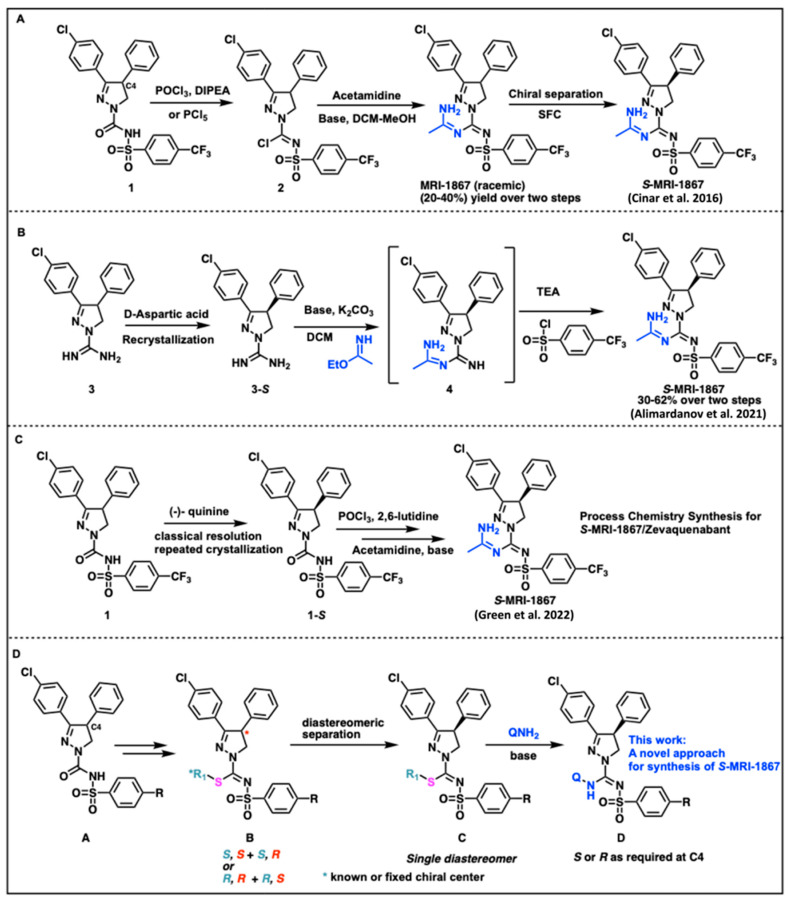
(**A**–**C**) Discovery-based, scalable, and process-chemistry based synthetic routes for *S*-MRI-1867 [27,43,44]; (**D**). Proposed novel synthetic approach towards *S*-MRI-1867.

Compounds like MRI-1867 or ibipinabant based on the pyrazoline heterocyclic core have a singular quaternary carbon stereocenter denoted as the C4 position and presents an interesting challenge (Figure 1). Previous methods have used chiral separation of the final racemic mixture to yield the *S*-enantiomer/eutomer. A racemic approach to MRI-1867 has been developed by Iyer et al. [27,28,37]. The discovery process allowed access to large numbers of analogs for SAR studies [28,42]. However, the unoptimized synthesis was low-yielding and included intractable conditions along with a need for supercritical fluid chromatography (SFC)/chiral prep LC based techniques (Figure 2A).

A high-yielding and scalable synthetic approach to provide substantial amounts of enantiomerically pure *S*-MRI-1867 for biological evaluation was reported in the patent literature [43,44]. Different approaches independently using classical resolution-based techniques was utilized in these methods. The groups used D-aspartic acid and (−)-quinine to generate intermediates that could be processed on to the active pharmaceutical ingredient (API) (Figure 2B,C). In our investigations with Bunte salts-based chemistry we reported a new class of compounds with sulfur embedding [45]. Subsequently, this approach illuminated a new, alternate route to generate chiral compounds of the type *S*-MRI-1867 as outlined in Figure 2D.

We envisioned a diastereomeric transform using an underexplored thioalkyl/thiofunctionalized group as a chiral auxiliary (Figure 2D). In order to access *S*-MRI-1867, we rationalized that sulfonyl urea **A** can be converted to a chiral thioalkyl bearing compound **B** containing a known stereocenter to obtain diastereomers of the type **B**. The diastereomeric mixture at this stage could potentially be separated under crystallization or flash purification conditions to give component diastereomers. Displacement of the unique thioalkyl group under controlled basic conditions can deliver the requisite enantiomer of type **D** in high enantiomeric purity.

**Scheme 1:**
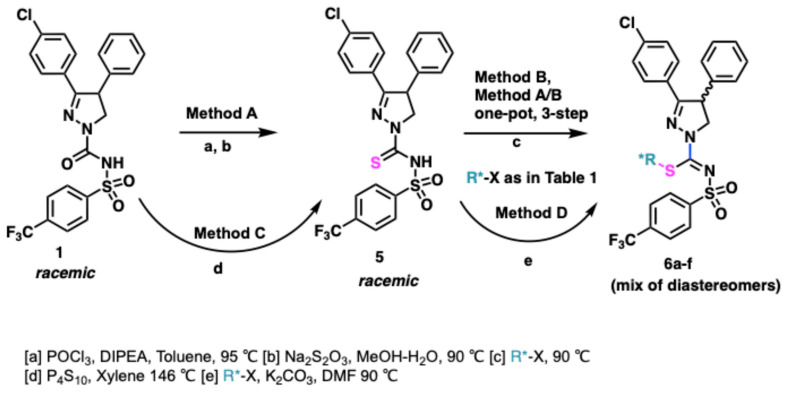
Synthesis protocol based on chiral thio derivatives.

This route was put to practice as shown in Scheme 1. Briefly the route was optimized to obtain sulfonyl thiourea **5** from the known sulfonyl urea **1** [45]. The diastereomeric compounds of type **6** could be generated by the alkylation of the sulfonyl thiourea **5** using a chiral alkylating agent with a known stereochemistry. Alternatively, sulfonyl urea compound **1** was converted to sulfonyl thiourea **5** using P_4_S_10_ by refluxing in xylene (Method C/Scheme 1). This method, however, required harsh conditions and extremely high temperatures to bring about the conversion of sulfonyl urea to sulfonyl thiourea. This clearly validated the preference for the utilization of milder, odor-free generation of sulfur analogs using the Bunte salt-based approach (Method A/Scheme 1).

Following the conversion of urea to thiourea, Bunte-salt method or simple base-mediated method allowed us access to thioalkyl compounds **6a**–**6f** with a known stereochemistry at the sulfur center (Table 1). The Bunte-salt method offered additional incentives in a sustainable approach using aqueous conditions and carrying out the chlorination, sulfur insertion, and alkylation in a one-pot/three-step protocol. In principle, various alkylating agents could be used to alkylate the thio center. A subset of compounds **6a**–**6f** were generated by this method. Compounds **6a**–**6e** analogs did not offer a clear separability of diastereomers under simple flash conditions or enrichment under crystallization conditions. However, when the sulfur center bore the S-propionate group using a commercial reagent methyl (*S*)-3-bromo-2-methylpropanoate, compound **6f** showed promising potential for the separation of diastereomers [45].

Compound **6f** was finally used as an example in its *S*-form at the sulfur center to bring about the transformation from compound **5** to the thioalkyl diastereomeric decoy intermediate for further transformations. This enabled the generation of compound **7** (*S*,*S* diastereomer) with a fixed stereochemistry at the thioalkyl center as detailed [45].

While TLC showed a modest separation of the diastereomeric **6f**, limited crystallization attempts showed that when compound **6f** was dissolved in hot ethanol and set aside for a few days, tiny crystals enriched in one diastereomer started to form. The stereochemistry of the diastereomers **6f** was assigned by working backwards where a small sample was separated on *R*,*R*-Whelk-O chiral column. The two diastereomers obtained as such were disparately treated with acetamidine in the presence of triethylamine to give *S*-MRI-1867 and *R*-MRI-1867. Knowing the stereochemistry at the *S*-alkylation center, the fraction that gave the *S*-MRI-1867 was assigned to arise from the *S*,*S*-diastereomer and the *R*-MRI-1867 product was from the *S*,*R*–diastereomer (*R* at the heterocyclic ring C4 position, compound **8**). The preferential crystallization process could thus also be expedited by treating compound **6f** mixture with seed crystals of the *S*,*S*-diastereomer obtained from chiral separation. The purported *S*,*S*-diastereomer compound **7** could be obtained in greater than 99% diastereomeric purity with at least 2 successive re-crystallizations from ethanol and a final IPA recrystallization avoiding chiral SFC in up to 38% yield from **6f**. Transformation of **7** to compound *S*-MRI-1867 could be achieved by treatment with acetamidine and Et_3_N in a DCM:MeOH mixture. The product obtained was confirmed as the desired *S* configuration product at the C4 center with 97.4% ee and greater than 95% purity by the analysis and comparison of all spectroscopic data with an authentic sample, which was previously purified by chiral HPLC/SFC and assigned as *S*-enantiomer by X-ray crystallography (see Supporting Information).

## 3. Conclusions

Our lab has developed a novel, improved protocol to access compounds of the type *S*-MRI-1867 without SFC based separation. Our current protocol is based on a unique Bunte-salt based method developed to generate thiocarboxamides. The thiocarboxamide can be appended in situ or stepwise with myriad alkylating groups. Judicious installation of alkylating agents with a predetermined stereocenter leads to an isothiourea-based chiral auxiliary/decoy intermediate to generate diastereomeric isothiourea precursors. This allows for chiral resolution of the C4 stereocenter on the dihydropyrazoline heterocyclic ring. The single diastereomer could then be displaced by amino pendants in a simple base-assisted reaction to give a chiral supercritical chromatography (SFC)-free approach to generate enantiomeric APIs. It is to be noted that no mercury-based reagents were needed for thio displacement [46]. The method provided validation of its orthogonal utility by enabling an expeditious assembly of chiral pyrazoline sulfonyl carboximdamide CB1 receptor antagonist/iNOS blocker, *S*-MRI-1867 [37,42] (zevaquenabant).

**Scheme 2:**
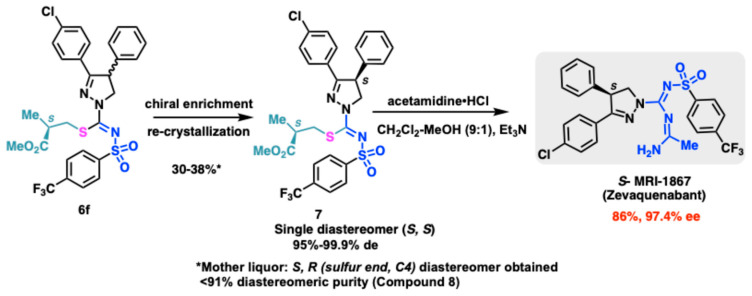
Application of the protocol in chiral synthesis of *S*-MRI-1867.

Compounds of the type *S*-MRI-1867 have a singular quaternary carbon stereocenter denoted as the C4 position of the pyrazoline ring. Previous methods have used chiral HPLC/SFC separation of the final racemic mixture to yield the biologically active *S*- enantiomer [28,46]. Alternatively, classical resolution methods have also been revealed in the patent literature. Since our new methodology was amenable to multi-gram scale synthesis of the sulfonylthiocarboxamide precursor **5**, we chose to synthesize the precursor of type **B** (via **5**) through an in situ chiral alkylating agent bearing a known stereocenter. This ensured access to a separable diastereomeric mixture which could afford a single diastereomer of the type **C**. Displacement of the thioalkyl group under controlled basic conditions can then deliver the requisite enantiomeric API (Scheme 2). The diastereomeric mixture **6f** thus enabled the generation of single diastereomer **7** through a preferential recrystallization.

To summarize, we have developed an effective approach to synthesize (*S*)-*N*-(1-aminoethylidene)-3-(4-chlorophenyl)-4-phenyl-*N*′-((4-(trifluoromethyl)phenyl)sulfonyl)-4,5-dihydro-1*H*-pyrazole-1-carboximidamide, a selective CB_1_ receptor inverse agonist with 86% yield for the final step and high ee (97.4%). Thus, this synthetic approach eliminates the need for SFC separation of the racemic mixture in the final step to yield the *S*-enantiomer of the lead compound *S*-MRI-1867. Currently *S*-MRI-1867 as INV1-01/zevaquenabant has completed Phase 1 clinical studies, and this novel synthetNic approach is being used in our lab for the further scale-up and optimization of similar compounds bearing the sulfonyl urea scaffold. Future work will enable us to generate a new library of compounds for expanded structure-function studies on the CB_1_R as peripheral antagonists for treatment of metabolic syndrome disorders.

## 4. Materials and Methods

Commercially available reagents were purchased and used as is. The starting materials were synthesized based on the literature procedures. NMR (^1^H NMR/^13^CNMR) spectra were recorded on a JEOL 400 MHz spectrometer (Akishima, Japan) in CDCl_3_ or DMSO-*d*_6_ (unless otherwise noted) with the values given in ppm (TMS as internal standard) and *J* (Hz) assignments of ^1^H resonance coupling. Time of Flight (TOF) High Resolution Mass spectra (HRMS) were recorded on a Waters Xevo G2 qTOF (Millford, MA, USA) or an Agilent ToF instrument (Santa Clara, CA, USA). Thin layer chromatography (TLC) analyses were carried out on prescored silica gel GHLF 0.25 mm plates (Miles Scientific, Newark, DE, USA) using various gradients of CHCl_3_/MeOH containing 1% NH_4_OH or gradients of EtOAc:*n*-hexane (30–50%). Visualization was accomplished under UV light or by staining in an iodine chamber. Flash column chromatography was performed on Teledyne ISCO Combiflash system (Lincoln, NE, USA) with and without PurIon mass detector (Lincoln, NE, USA). For reactions that required heating, commercially available heating block, pie-block, and stir-plate were used. Product yields are un-optimized unless indicated. All compounds characterized had ≥95% purity unless indicated. Purity and structural confirmation were analyzed by a combination of TLC, ^1^H-NMR, ^13^C-NMR (where available), LC/MS (available upon request), high-resolution mass spec and X-ray (as indicated). LC-MS detection was carried out on Agilent 1200 using Poroshell 120 EC-C18, (3 × 50 mm/2.7 μM). The mobile phase was 50% to 98% acetonitrile (1% formic acid) over 8 min standard gradient. The LC-MS chromatogram showed the correct molecular (MH^+^) ion as well as a single peak at UV (254 nm). Chiral HPLC was carried using Whelko (*R*,*R*) (Regis Technolgies Inc, Morton Grove, IL, USA) chiral column using method as indicated for specific compounds.

**3-(4-Chlorophenyl)-4-phenyl-*N*-((4-(trifluoromethyl)phenyl)sulfonyl)-4,5-dihydro-1*H*-pyrazole-1-carboxamide (1)** [45,46]

### 4.1. Method A [45]

**3-(4-Chlorophenyl)-4-phenyl-*N*-((4-(trifluoromethyl)phenyl)sulfonyl)-4,5-dihydro-1*H*-pyrazole-1-carbothioamide (5):**

As previously reported, POCl_3_ (1.5 mmol) was added to a mixture of compound **1** (508 mg, 1.0 mmol) in toluene (5 mL), followed by the addition of *N*,*N*-diisopropylethylamine (DIPEA) (1.5 mmol) and the mixture was heated at 95 °C for 1.5 h under N_2_ atmosphere. The reaction mixture was then cooled, and the excess reagents in toluene were evaporated in vacuo. The imidoyl chloride intermediate was dissolved in methanol (5 mL); sodium thiosulfate (2 eq, 316 mg, 2.0 mmol) dissolved in 0.5 mL water was added, dropwise, to this solution and then the reaction was heated to 90 °C. Upon completion of the reaction, as seen by TLC, the reaction mixture was cooled to room temperature and methanol was evaporated. The slurry was triturated with isopropyl alcohol to obtain white slurry. The solution was filtered and washed with a mixture of cold IPA, and water to give compound **5** as a white fluffy solid. (490 mg, 94% yield). ^1^H-NMR (400 MHz, CDCl_3_): δ ^1^H-NMR (400 MHz, CDCl_3_): δ 9.64 (s, 1H), 8.30 (d, *J* = 8.0 Hz, 2H), 7.81 (d, *J* = 8.1 Hz, 2H), 7.60 (d, *J* = 8.5 Hz, 2H), 7.31 (d, *J* = 7.6 Hz, 5H), 7.11 (d, *J* = 6.7 Hz, 2H), 4.77 (dd, *J* = 11.1, 5.0 Hz, 1H), 4.58 (t, *J* = 12.1 Hz, 1H), 4.19 (dd, *J* = 12.8, 4.7 Hz, 1H). ^13^C-NMR (101 MHz, CDCl_3_): δ 169.08, 159.44, 142.01, 138.73, 137.72, 135.48, 135.16, 130.02, 129.78, 129.33, 129.25, 128.42, 127.57, 127.34, 125.88, 125.84, 125.81, 124.60, 58.01, 51.21. MH^+^ LRMS 524.1, HRMS (ESI) *m*/*z*: [M + H]^+^ Calcd for (C_23_H_18_ClN_3_O_2_F_3_S_2_) 524.0481; Found 524.0480.

### 4.2. Method B [45]

**General procedure for in situ alkylation of thio-sulfonyl ureas.**

Following Method A, upon completion of reaction as seen by TLC/LCMSF, the respective alkylating agent (corresponding to **6a**/**6f**) was added dropwise to the thiocarboxamide, obtained from Method A, reaction mixture and the reaction continued until all the thiourea was consumed as seen by TLC/LCMS. The reaction was cooled to room temperature and methanol was evaporated. The organic mixture was then extracted into dichloromethane, washed with brine, and dried over Na_2_SO_4_. The solvent was removed in vacuo and dried thoroughly to afford a sticky solid. The sticky solid was purified by flash chromatography (20% hexanes in EtOAc) to give thio-alkylated sulfonyl compounds.

### 4.3. Method C

**3-(4-Chlorophenyl)-4-phenyl-*N*-((4-(trifluoromethyl)phenyl)sulfonyl)-4,5-dihydro-1*H*-pyrazole-1-carbothioamide** (**5**)

A sealed tube plus stir bar was charged with sulfonyl urea **1** (0.5 g, 1 mmol) and 10 mL xylene. P_4_S_10_ (0.22 g, 1.0 mmol) was added, and the reaction was then heated to 145 °C with stirring for 5 h. The reaction mixture was then allowed to cool to room temperature and stand overnight. The xylene solution was then filtered and washed with water. The filtrate was dried and collected to give a pale, yellow powder **5**. Yield (0.4 g, 76%).

^1^H-NMR (400 MHz, CDCl_3_): δ 9.64 (s, 1H), 8.30 (d, *J* = 8.0 Hz, 2H), 7.81 (d, *J* = 8.1 Hz, 2H), 7.60 (d, *J* = 8.5 Hz, 2H), 7.31 (d, *J* = 7.6 Hz, 5H), 7.11 (d, *J* = 6.7 Hz, 2H), 4.77 (dd, *J* = 11.1, 5.0 Hz, 1H), 4.58 (t, *J* = 12.1 Hz, 1H), 4.19 (dd, *J* = 12.8, 4.7 Hz, 1H). ^13^C-NMR (101 MHz, CDCl_3_): δ 169.08, 159.44, 142.01, 138.73, 137.72, 135.48, 135.16, 130.02, 129.78, 129.33, 129.25, 128.42, 127.57, 127.34, 125.88, 125.84, 125.81, 124.60, 58.01, 51.21. MH^+^ LRMS 524.1, HRMS (ESI) *m*/*z*: [M + H]^+^ Calcd for (C_23_H_18_ClN_3_O_2_F_3_S_2_) 524.0481; Found 524.0480.

### 4.4. Method D

**General procedure for step-wise alkylation of thio -sulfonyl ureas.**

(−)-Methyl (*S*)-3-bromo-2-methylpropionate (0.35 mL, 2.7 mmol) and K_2_CO_3_ (0.37 g, 2.7 mmol) were added to a solution of compound **5** obtained from Method A or C (0.5 g, 1.0 mmol) in DMF (10 mL) at room temperature. Upon completion of the reaction, the reaction mixture was partitioned between ether and water. The organic layer was washed with water and dried. Purification on a silica gel column using hexanes-ethyl acetate (3:1) yielded compound **3** as a pale yellow sticky solid (0.31 g, 57%).

The synthesis of alkylating agents used in **6b**–**6e** was carried our as previously reported.

**(*S*)-2-Bromo-*N*-(3-oxobutan-2-yl)acetamide** [47,48]

**(*S*)-2-Bromo-*N*-(2-methyl-4-oxopentan-3-yl)acetamide (valinate)** [48]

**(*S*)-1-(2-Acetylpyrrolidin-1-yl)-2-bromoethan-1-one** [49]

**(*R*)-2-Bromo-*N*-(1-phenylethyl)acetamide** [50]

**(*S*)-2-Methylbutyl (*Z*)-3-(4-chlorophenyl)-4-phenyl-*N*-((4-(trifluoromethyl)phenyl)sulfonyl)-4,5-dihydro-1*H*-pyrazole-1-carbimidothioate (6a)**

POCl_3_ (3 mmol) was added to a mixture of sulfonylurea compound **1** (100 mg, 0.2 mmol) in toluene (5 mL), followed by the addition of *N*,*N*-diisopropylethylamine (DIPEA) (3.0 mmol) and the mixture was heated to 95 °C for 1.5 h under N_2_ atmosphere. The reaction mixture was then cooled, and the excess reagents in toluene were evaporated in vacuo. The imidoyl chloride intermediate was dissolved in methanol (7 mL), sodium thiosulfate (2 eq, dissolved in 0.5 mL water was added dropwise to this solution, and the reaction was heated to 90 °C. Upon completion of the reaction, as seen by TLC/LCMS, (*S*)-1-bromo-2-methylbutane was added dropwise to the cooled reaction mixture and the reaction continued until all the thiourea was consumed, as seen by TLC/LCMS. The reaction was cooled to room temperature and methanol was evaporated. The organic mixture was the extracted into dichloromethane, washed with brine, and dried over Na_2_SO_4_. The solvent was removed in vacuo and dried thoroughly to afford a yellow solid. This solid was purified by flash chromatography (30% hexanes in EtOAc) to give thio-alkylated sulfonyl compound (**6a**) as a pale yellow solid (mix of diastereomers) (Yield 79 mg, 67%).

^1^H-NMR (400 MHz, CDCl_3_): δ 8.08 (d, *J* = 7.8 Hz, 3H), 7.73 (d, *J* = 7.8 Hz, 3H), 7.61 (d, *J* = 7.7 Hz, 3H), 7.32–7.27 (m, 6H), 7.16 (d, *J* = 6.9 Hz, 3H), 4.94 (s, 1H), 4.83–4.80 (m, 2H), 4.50 (s, 1H), 2.85 (td, *J* = 13.6, 5.2 Hz, 1H), 2.61 (dt, *J* = 13.5, 7.1 Hz, 1H), 1.51 (d, *J* = 5.3 Hz, 1H), 1.21 (t, *J* = 9.2 Hz, 2H), 1.04 (dd, *J* = 14.0, 7.0 Hz, 1H), 0.80 (d, *J* = 6.4 Hz, 4H), 0.69 (t, *J* = 7.2 Hz, 4H). ^13^C-NMR (101 MHz, CDCl_3_): δ 160.05, 160.03, 159.98, 147.49, 147.45, 138.67, 137.27, 133.55, 133.22, 129.76, 129.69, 129.35, 129.25, 129.14, 128.37, 127.99, 127.62, 127.44, 127.19, 126.82, 126.74, 125.84, 125.81, 125.77, 125.74, 124.91, 122.20, 119.49, 60.13, 52.22, 52.20, 40.09, 40.03, 34.59, 28.80, 28.73, 18.54, 18.51, 11.11. MH^+^ LRMS 5941, HRMS (ESI) *m*/*z*: [M + H]^+^ Calcd for (C_28_H_28_ClN_3_O_2_F_3_S_2_) 594.1258; Found 594.1253.

**Methyl (2-(((*Z*)-(3-(4-chlorophenyl)-4-phenyl-4,5-dihydro-1*H*-pyrazol-1-yl)(((4-(trifluoromethyl)phenyl)sulfonyl)imino)methyl)thio)acetyl)-*L*-alaninate (6b)**

(*S*)-2-Bromo-*N*-(3-oxobutan-2-yl)acetamide [47,48] (158 mg, 0.76 mmol) and K_2_CO_3_ (209 mg, 1.52 mmol) were added to a solution of compound **5** (200 mg, 0.38 mmol) in DMF (10 mL) at room temperature. Upon completion of the reaction, the reaction mixture was partitioned between ether and water. The organic layer was washed with water and dried. Purification on a silica gel column using hexanes-ethyl acetate (3:1) yielded compound **6b** as a pale yellow sticky solid (97 mg, 38%) (mix of diastereomers).

^1^H-NMR (400 MHz, CDCl_3_): δ 8.07 (d, *J* = 7.7 Hz, 4H), 7.74 (d, *J* = 7.8 Hz, 4H), 7.58 (d, *J* = 7.8 Hz, 4H), 7.30 (dd, *J* = 15.3, 6.9 Hz, 8H), 7.14 (d, *J* = 6.5 Hz, 4H), 4.93 (d, *J* = 10.3 Hz, 1H), 4.82 (d, *J* = 12.0 Hz, 3H), 4.48 (dd, *J* = 15.0, 7.5 Hz, 3H), 3.75–3.58 (m, 9H), 1.35 (t, *J* = 7.4 Hz, 6H). ^13^C-NMR (101 MHz, CDCl_3_): δ 172.97, 172.92, 166.80, 166.76, 161.48, 161.33, 159.50, 159.28, 146.31, 138.48, 138.32, 137.65, 129.81, 129.71, 129.43, 129.39, 129.22, 128.47, 128.39, 127.66, 127.63, 127.53, 127.45, 127.08, 127.03, 126.03, 125.99, 125.96, 124.81, 122.10, 60.09, 60.07, 60.05, 59.71, 59.69, 52.57, 52.45, 52.23, 52.08, 48.55, 48.49, 36.65, 36.49, 29.79, 17.89, 17.80. MH^+^ LRMS 667.1, HRMS (ESI) *m*/*z*: [M + H]^+^ Calcd for (C_29_H_27_ClN_4_O_5_F_3_S_2_) 667.1058; Found 667.1052.

**Methyl (2-(((*Z*)-(3-(4-chlorophenyl)-4-phenyl-4,5-dihydro-1*H*-pyrazol-1-yl)(((4-(trifluoromethyl)phenyl)sulfonyl)imino)methyl)thio)acetyl)-*L*-valinate (6c)**

(*S*)-2-Bromo-*N*-(2-methyl-4-oxopentan-3-yl)acetamide [48] (179 mg, 0.76 mmol) and K_2_CO_3_ (209 mg, 1.52 mmol) were added to a solution of compound **5** (200 mg, 0.38 mmol) in DMF (10 mL) at room temperature. Upon completion of the reaction, the reaction mixture was partitioned between ether and water. The organic layer was washed with water and dried. Purification on a silica gel column using hexanes-ethyl acetate (3:1) yielded compound **6c** as a pale white sticky solid (114 mg, 43%) (mix of diastereomers).

^1^H-NMR (400 MHz, CDCl_3_): δ 8.08 (s, 2H), 7.76–7.75 (m, 2H), 7.59 (d, *J* = 7.2 Hz, 2H), 7.33–7.29 (m, 5H), 7.14–7.00 (m, 3H), 5.00 (s, 1H), 4.85–4.81 (m, 1H), 4.55–4.51 (m, 1H), 4.42–4.39 (m, 1H), 3.70–3.62 (m, 5H), 2.11–2.03 (m, 1H), 1.24 (s, 7H), 0.86–0.83 (m, 8H). ^13^C-NMR (101 MHz, CDCl_3_): ^13^C-NMR (101 MHz, CDCl_3_): δ 172.05, 167.40, 167.19, 138.38, 138.35, 137.73, 137.68, 129.84, 129.80, 129.48, 129.44, 129.24, 128.51, 128.46, 127.59, 127.54, 127.40, 127.38, 127.04, 126.07, 126.03, 125.99, 57.72, 52.50, 52.38, 52.27, 52.20, 36.33, 36.16, 31.17, 31.12, 19.04, 18.11, 18.06.MH^+^ LRMS 695.1, HRMS (ESI) *m*/*z*: [M + H]^+^ Calcd for (C_31_H_31_ClN_4_O_5_F_3_S_2_) 695.1371; Found 695.1367.

**Methyl (2-(((*Z*)-(3-(4-chlorophenyl)-4-phenyl-4,5-dihydro-1*H*-pyrazol-1-yl)(((4-(trifluoromethyl)phenyl)sulfonyl)imino)methyl)thio)acetyl)-*L*-prolinate (6d)**

(*S*)-1-(2-Acetylpyrrolidin-1-yl)-2-bromoethan-1-one (177 mg, 0.76 mmol) and K_2_CO_3_ (209 mg, 1.52 mmol) were added to a solution of compound **5** (200 mg, 0.38 mmol) in DMF (10 mL) at room temperature. Upon completion of the reaction, the reaction mixture was partitioned between ether and water. The organic layer was washed with water and dried. Purification on a silica gel column using hexanes-ethyl acetate (3:1) yielded compound **6d** as a yellow sticky solid (98 mg, 37%) (mix of diastereomers).

^1^H-NMR (400 MHz, CDCl_3_): δ 8.08 (d, *J* = 7.7 Hz, 2H), 7.74 (d, *J* = 7.6 Hz, 2H), 7.58 (d, *J* = 7.7 Hz, 2H), 7.29 (t, *J* = 7.6 Hz, 3H), 7.23 (s, 1H), 7.15 (s, 2H), 4.97 (d, *J* = 1.1 Hz, 1H), 4.84 (d, *J* = 10.1 Hz, 1H), 4.51 (dd, *J* = 4.5, 2.0 Hz, 1H), 4.36 (d, *J* = 6.9 Hz, 1H), 3.68 (q, *J* = 12.2 Hz, 4H), 3.27 (s, 1H), 3.09 (s, 1H), 1.93 (t, *J* = 51.0 Hz, 4H). ^13^C-NMR (101 MHz, CDCl_3_): δ 172.36, 165.52, 165.47, 160.43, 160.37, 138.52, 137.42, 129.77, 129.46, 129.12, 128.40, 127.74, 127.71, 127.58, 127.50, 127.48, 127.13, 126.94, 126.84, 125.85, 125.81, 124.86, 122.15, 59.15, 52.44, 46.95, 46.93, 36.60, 36.55, 31.33, 29.79, 29.14, 24.69, 22.53.

MH^+^ LRMS 693.1, HRMS (ESI) *m*/*z*: [M + H]^+^ Calcd for (C_31_H_29_ClN_4_O_5_F_3_S_2_) 693.1582; Found 693.175.

**2-Oxo-2-(((*R*)-1-phenylethyl)amino)ethyl (*Z*)-3-(4-chlorophenyl)-4-phenyl-*N*-((4-(trifluoromethyl)phenyl)sulfonyl)-4,5-dihydro-1*H*-pyrazole-1-carbimidothioate (6e)**

(*R*)-2-Bromo-*N*-(1-phenylethyl)acetamide [50] (184 mg, 0.76 mmol) and K_2_CO_3_ (209 mg, 1.52 mmol) were added to a solution of compound **5** (200 mg, 0.38 mmol) in DMF (10 mL) at room temperature. Upon completion of the reaction, the reaction mixture was partitioned between ether and water. The organic layer was washed with water and dried. Purification on a silica gel column using hexanes-ethyl acetate (3:1) yielded compound **6e** as a white solid (132 mg, 51%) (mix of diastereomers).

**^1^H-NMR** (400 MHz, CDCl_3_): δ 7.95 (d, *J* = 7.8 Hz, 2H), 7.90 (d, *J* = 7.7 Hz, 2H), 7.67 (t, *J* = 7.8 Hz, 4H), 7.56 (d, *J* = 7.7 Hz, 4H), 7.30 (t, *J* = 12.4 Hz, 13H), 7.12 (d, *J* = 4.5 Hz, 5H), 7.06–7.05 (m, 2H), 5.06 (s, 2H), 4.76–4.72 (m, 3H), 4.64 (t, *J* = 11.9 Hz, 1H), 4.32–4.29 (m, 1H), 4.14 (d, *J* = 10.7 Hz, 1H), 3.97–3.93 (m, 1H), 3.75 (s, 2H), 3.65 (d, *J* = 16.2 Hz, 1H), 1.46 (d, *J* = 6.6 Hz, 3H), 1.43 (d, *J* = 6.9 Hz, 3H).

^13^C-NMR (101 MHz, CDCl_3_): δ 166.00, 161.51, 159.57, 159.53, 143.17, 138.37, 138.12, 137.73, 129.86, 129.41, 129.36, 129.25, 128.72, 128.58, 128.53, 128.46, 127.56, 127.48, 127.37, 127.32, 127.29, 127.00, 126.48, 126.38, 126.05, 126.01, 125.97, 52.01, 51.93, 49.28, 36.87, 36.83, 36.81, 21.62. MH^+^ LRMS 685.0, HRMS (ESI) *m*/*z*: [M + H]^+^ Calcd for (C_33_H_29_ClN_4_O_3_F_3_S_2_) 685.1316; Found 685.1320.

**Methyl (*S*)-(3-(((3-(4-chlorophenyl)-4-phenyl-4,5-dihydro-1*H*-pyrazol-1-yl)(((4-(trifluoro-methyl)phenyl)sulfonyl)imino)methyl) thio)-2-methylpropanoate (6f)**

(−)-Methyl (*S*)-3-bromo-2-methylpropionate (0.35 mL, 2.7 mmol) and K_2_CO_3_ (0.37 g, 2.7 mmol) were added to a solution of compound **2** (0.94 g, 1.8 mmol) in DMF (10 mL) at room temperature. Upon completion of the reaction, the reaction mixture was partitioned between ether and water. The organic layer was washed with water and dried. Purification on a silica gel column using hexanes-ethyl acetate (3:1) yielded compound **3** as a pale yellow sticky solid (0.63 g, 57%) (mix of diastereomers).

^1^H-NMR (400 MHz, CDCl_3_): δ 8.10 (d, *J* = 7.3 Hz, 3H), 7.76 (d, *J* = 7.8 Hz, 3H), 7.61 (d, *J* = 6.6 Hz, 3H), 7.33 (d, *J* = 6.8 Hz, 4H), 7.28 (s, 2H), 7.18 (s, 3H), 5.10–5.00 (m, 1H), 4.87–4.84 (m, 2H), 4.64–4.55 (m, 1H), 3.61 (d, *J* = 4.5 Hz, 5H), 3.11–3.03 (m, 1H), 2.81 (t, *J* = 6.6 Hz, 1H), 2.63 (t, *J* = 5.9 Hz, 1H), 0.96 (d, *J* = 5.7 Hz, 5H).

^13^C-NMR (101 MHz, CDCl_3_): δ 175.14, 160.18, 138.54, 137.41, 129.79, 129.43, 129.16, 128.43, 127.79, 127.48, 127.45, 126.76, 125.90, 125.86, 52.56, 52.02, 39.56, 39.49, 35.26, 29.80, 16.93). MH^+^ LRMS 624.1, HRMS (ESI) *m*/*z*: [M + H]^+^ Calcd for (C_28_H_26_ClN_3_O_4_F_3_S_2_) 624.1005; Found *m*/*z* 624.1006. (*R*,*R*)-Whelk-O1 chiral column (250 mm × 4.6 mm/5 μm): 100% EtOH 1.5 mL/min (254 nm) Peak 1 = 3.95 min, Peak 2 = 5.03 min.

Alternatively, Compound **1** (100 mg, 0.2 mmol) using Method A and Method B can be converted to compound **6f** (yield 78 mg, 63% over 3 steps).

**(*S*,*S*** **)-Methyl(3-(3-(4-chlorophenyl)-4-phenyl-4,5-dihydro-1*H*-pyrazol-1-yl)(((4-(trifluoro-methyl)phenyl)sulfonyl)imino)methyl)thio)-2-methylpropanoate (7)**

Compound **6f,** upon slow crystallization in hot ethanol, showed enrichment in one of the enantiomers. This preferential crystallization of the diastereomeric compound could be accelerated by generating seed crystals from chiral separation of a small sample of **6f** on (*R*,*R*)-Whelk O chiral column. This diastereomer as shown previously by X-Ray crystallography showed up as *S*,*S*-diastereomer which was analyzed by way of fixed S-stereocenter at the sulfur appendage. The *S*,*S*-diastereomer was further obtained from successive crystallizations twice from ethanol (90–94% de), followed by a final isopropyl alcohol recrystallization to afford the compound **7** (with de up to 99% de).

^1^H-NMR (400 MHz, CDCl_3_): δ 8.08 (d, *J* = 7.8 Hz, 2H), 7.75 (d, *J* = 7.9 Hz, 2H), 7.60 (d, *J* = 7.7 Hz, 2H), 7.32 (d, *J* = 7.0 Hz, 6H), 7.16 (d, *J* = 6.8 Hz, 2H), 5.05 (s, 1H), 4.85 (dd, *J* = 10.2, 4.3 Hz, 1H), 4.61–4.59 (m, 1H), 3.60 (s, 3H), 3.07–3.02 (m, 1H), 2.82–2.78 (m, 1H), 2.64–2.59 (m, 1H), 0.95 (d, *J* = 6.8 Hz, 3H). ^13^C-NMR (101 MHz, CDCl_3_): δ 175.15, 138.53, 137.43, 129.86, 129.80, 129.43, 129.16, 128.44, 127.79, 127.45, 126.76, 125.94, 125.91, 125.87, 125.83, 52.57, 52.02, 39.56, 35.25, 16.94. MH^+^ LRMS 624.1, HRMS (ESI) *m*/*z*: [M + H]^+^ Calcd for (C_28_H_26_ClN_3_O_4_F_3_S_2_) 624.1005; Found *m*/*z* 624.1006. (*R*,*R*)-Whelk-O1 chiral column (250 mm × 4.6 mm/5 μm): 100% EtOH, 1.5 mL/min (254 nm) Peak 1 = 3.96 min.

**(*S*,*R*** **)-Methyl(3-(3-(4-chlorophenyl)-4-phenyl-4,5-dihydro-1*H*-pyrazol-1-yl)(((4-(trifluoro-methyl)phenyl)sulfonyl)imino)methyl)thio)-2-methylpropanoate (8)**

The *S*,*R*-diastereomer was further obtained from the mother liquor of successive crystallizations batches used to obtain **7**. Compound **8** could be obtained in up to 90% diastereomeric purity from ethanol-IPA mother liquor fractions following the filtration of compound **7**.

^1^H-NMR (400 MHz, CDCl_3_): δ 8.08 (d, *J* = 7.7 Hz, 2H), 7.75 (d, *J* = 7.8 Hz, 2H), 7.60 (d, *J* = 7.5 Hz, 2H), 7.33–7.27 (m, 6H), 7.17 (d, *J* = 6.6 Hz, 2H), 5.06 (s, 1H), 4.85 (dd, *J* = 5.7, 4.7 Hz, 1H), 4.61 (s, 1H), 3.61 (s, 3H), 3.05 (s, 1H), 2.79 (dd, *J* = 13.1, 5.1 Hz, 1H), 2.63 (d, *J* = 6.7 Hz, 1H), 0.96 (d, *J* = 6.5 Hz, 3H). ^13^C-NMR (101 MHz, CDCl_3_): δ 175.14, 138.54, 137.41, 129.79, 129.42, 129.16, 128.43, 127.79, 127.48, 126.76, 125.90, 125.86, 125.83, 52.03, 39.49, 35.30, 16.94. MH^+^ LRMS 624.1, HRMS (ESI) *m*/*z*: [M + H]^+^ Calcd for (C_28_H_26_ClN_3_O_4_F_3_S_2_) 624.1005; Found *m*/*z* 624.1006. (*R*,*R*)-Whelk-O1 chiral column (250 mm × 4.6 mm/5 μm): 100% EtOH, 1.5 mL/min (254 nm) Peak 1 = 5.2 min.

**(*S*)-*N*-(1-Aminoethylidene)-3-(4-chlorophenyl)-4-phenyl-*N*′-((4-(trifluoromethyl)phenyl)-sulfonyl)-4,5-dihydro-1*H*-pyrazole-1-carboximidamide (*S*-MRI-1867)**

Acetamidine•HCl (12 mg, 0.128 mmol) dissolved in DCM:MeOH (9:1) was added to a solution of Compound **7** in DCM (5 mL) (40 mg, 0.064 mmol) cooled to 0 °C, followed by Et_3_N (18 μL, 0.128 mmol). The reaction was warmed to room temperature and allowed to run until completion of the reaction (36–48 h). The organic layer was then extracted in DCM washed with water and dried over Na_2_SO_4_. Purification on a silica gel column using hexanes-ethyl acetate (1:1) yielded compound *S*-MRI-1867 as a white solid (30 mg, 86%). The compound was confirmed as the desired product with 97.4% ee by the comparison of all spectroscopic data with authentic sample, which was previously purified by chiral HPLC and assigned by X-ray Crystallography.

^1^H-NMR (400 MHz, CDCl_3_): δ 8.04 (d, *J* = 7.5 Hz, 2H), 7.68 (d, *J* = 7.8 Hz, 2H), 7.49 (d, *J* = 8.0 Hz, 2H), 7.28 (d, *J* = 8.1 Hz, 3H), 7.19 (s, 2H), 7.07 (d, *J* = 6.4 Hz, 2H), 5.18 (d, *J* = 0.5 Hz, 2H), 4.73–4.69 (m, 1H), 4.49 (t, *J* = 12.2 Hz, 1H), 4.12–4.08 (m, 1H), 2.08 (s, 3H). ^13^C-NMR (101 MHz, CDCl_3_): MH^+^ LRMS 548.1, HRMS (C_25_H_22_ClF_3_N_5_O_2_S) [M + H]^+^: calc 548.1129 found: 548.1132.

(*R*,*R*)-Whelk-O1 chiral column (250 mm × 4.6 mm/5 μm): 100% EtOH, 1 mL/min (254 nm) Peak 1 = 4.1 min.

## Figures and Tables

**Table 1:** Evaluation of R*-X groups for chiral enrichment.

Compound	R*-X	Conditions	Yield ^a^
**6a**	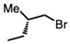	Method A, B	67%
**6b**	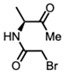	Method A, D	38%
**6c**	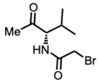	Method A, D	43%
**6d**	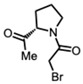	Method A, D	37%
**6e**	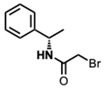	Method A, D	51%
**6f**	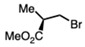	Method A, B	63%
**6f**	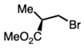	Method A, D	57%

^a^ For entry **6a**/**6f**, using Method A/B the yields are reported over 3-steps. For **6f** using method A/D For entries, the yields reported are for step e from compound **1**. For **6b**–**6e**, the yields reported are for step e from compound **5**.

## Data Availability

The original contributions presented in this study are included in the article and Supplementary Material. Further inquiries can be directed to the corresponding author.

## Supplementary Materials

The following supporting information can be downloaded at: https://www.mdpi.com/article/10.3390/molecules31030515/s1.
