# Supplementary material for: A Novel Approach for the Synthesis of Peripherally Acting Dual Target Inhibitor of Cannabinoid-1 (CB1 Receptor) and Inducible Nitric Oxide Synthase (iNOS) (S-MRI-1867/Zevaquenabant)
Source: Molecules. 2026 Feb 2;31(3):515. doi: 10.3390/molecules31030515 (PMC12899878; doi:10.3390/molecules31030515)
Supplement: Supplementary file 1 [file molecules-31-00515-s001.zip › molecules-4098725-supplementary.pdf]

A Novel Approach for the Synthesis of Peripherally Acting Dual Target Inhibitor of Cannabinoid-1 (CB1 Receptor) and Inducible Nitric Oxide Synthase (iNOS) (*S*-MRI-1867/Zevaquenabant)

Malliga R. Iyer

Section on Medicinal Chemistry, National Institute on Alcohol Abuse and Alcoholism, National Institutes of Health, 5625 Fishers Lane, Rockville, MD 20852, USA.

Supporting Data:

^{1}H-NMR and ^{13}C-NMR of Compound **5**

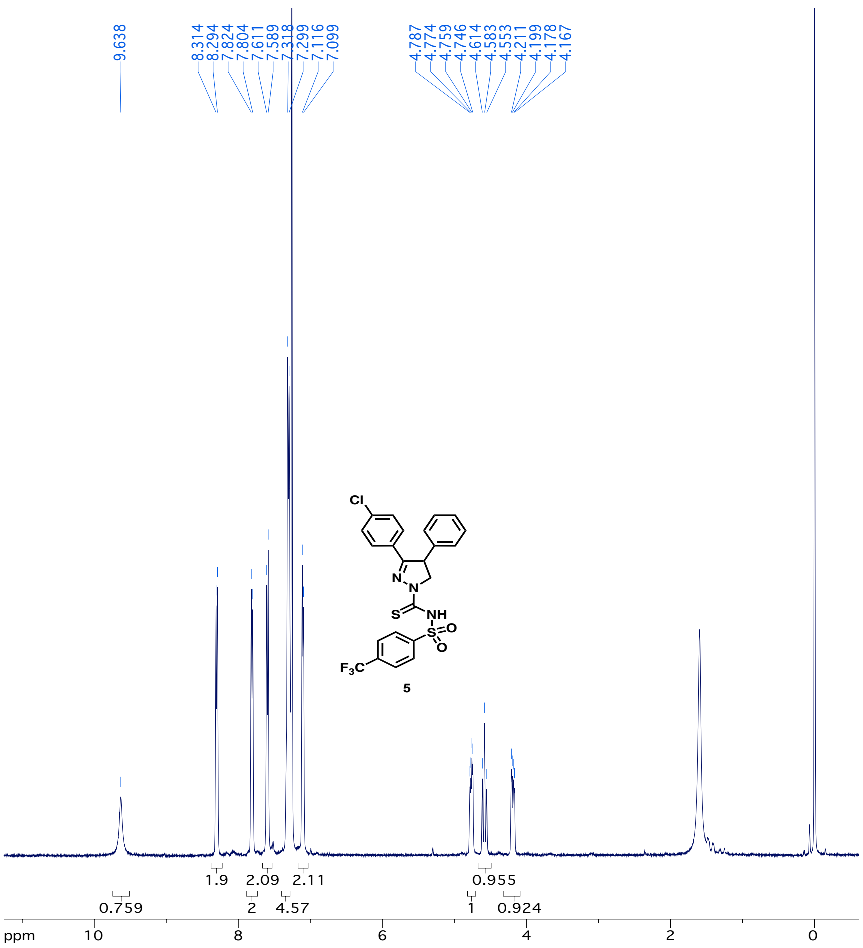

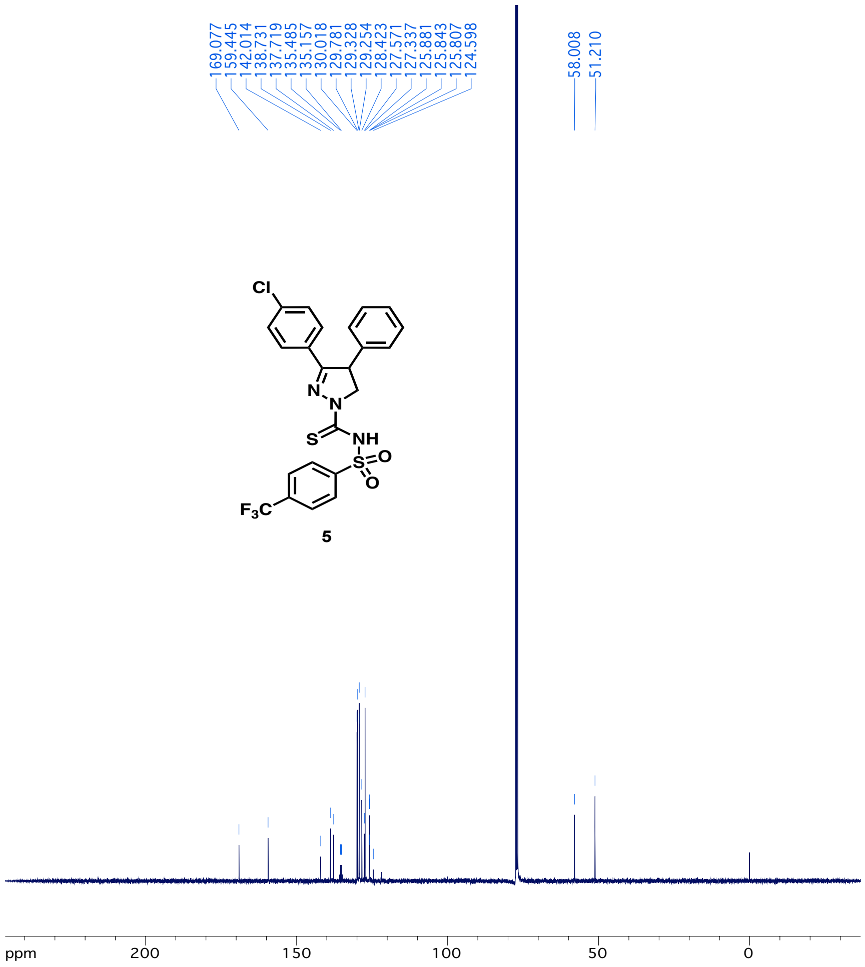

^{1}H-NMR and ^{13}C-NMR of Compound **6a**

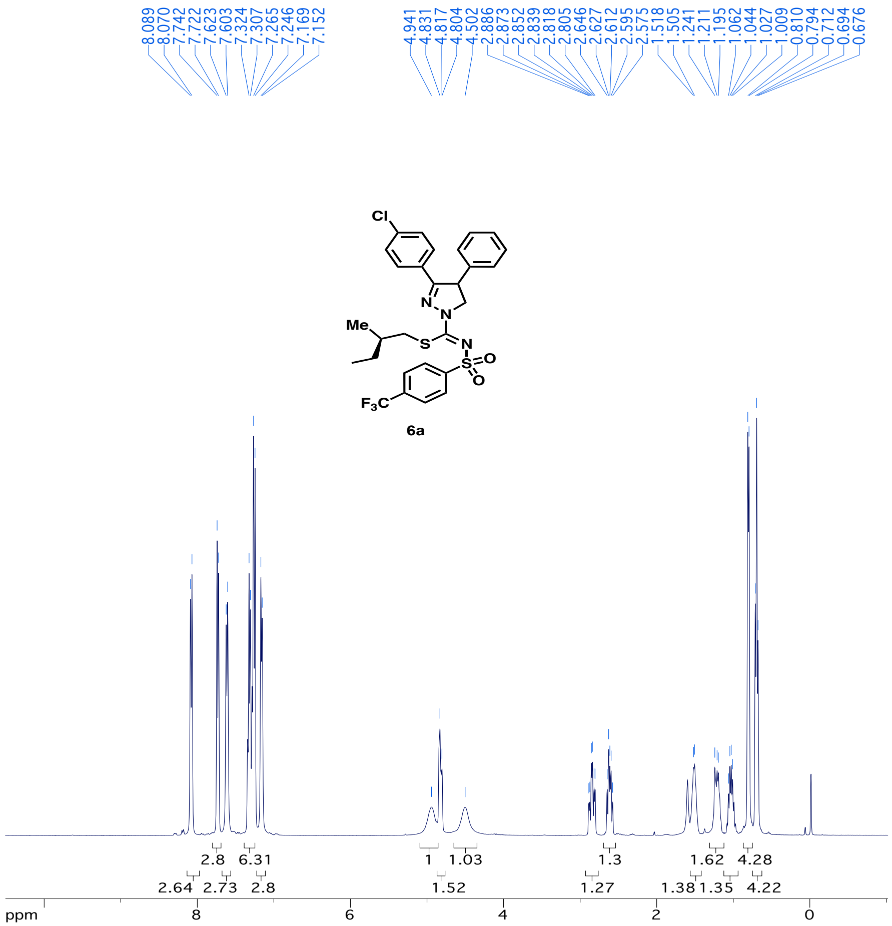

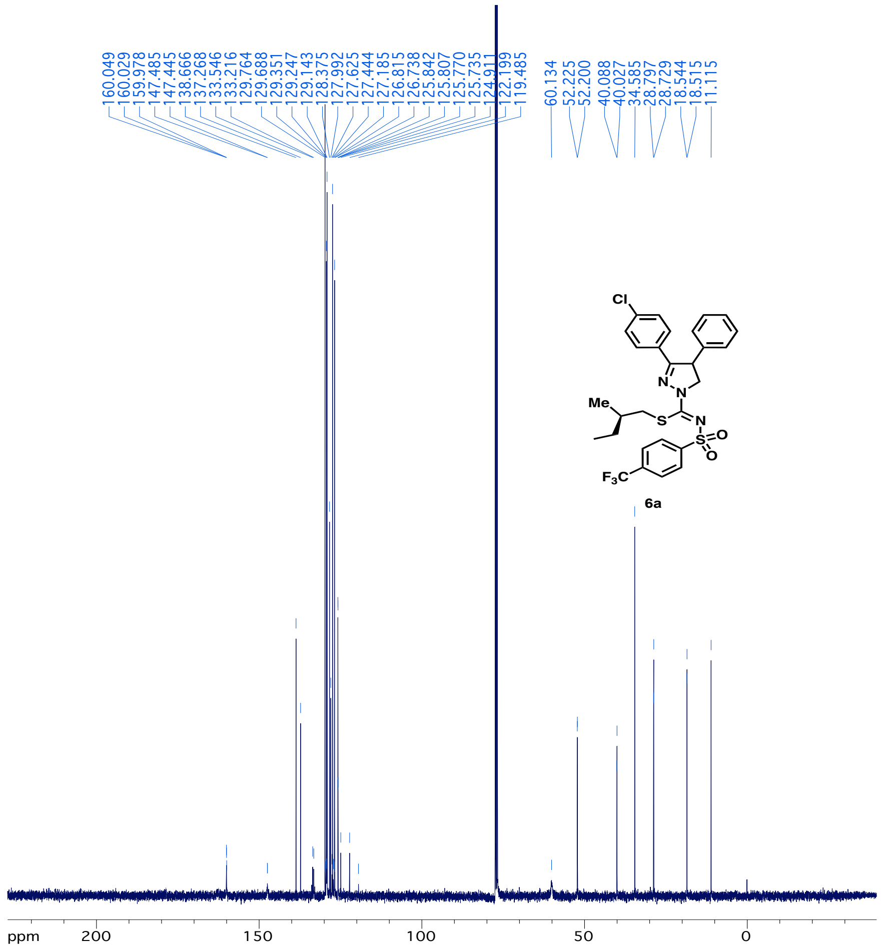

^{1}H-NMR and ^{13}C-NMR of Compound **6b**

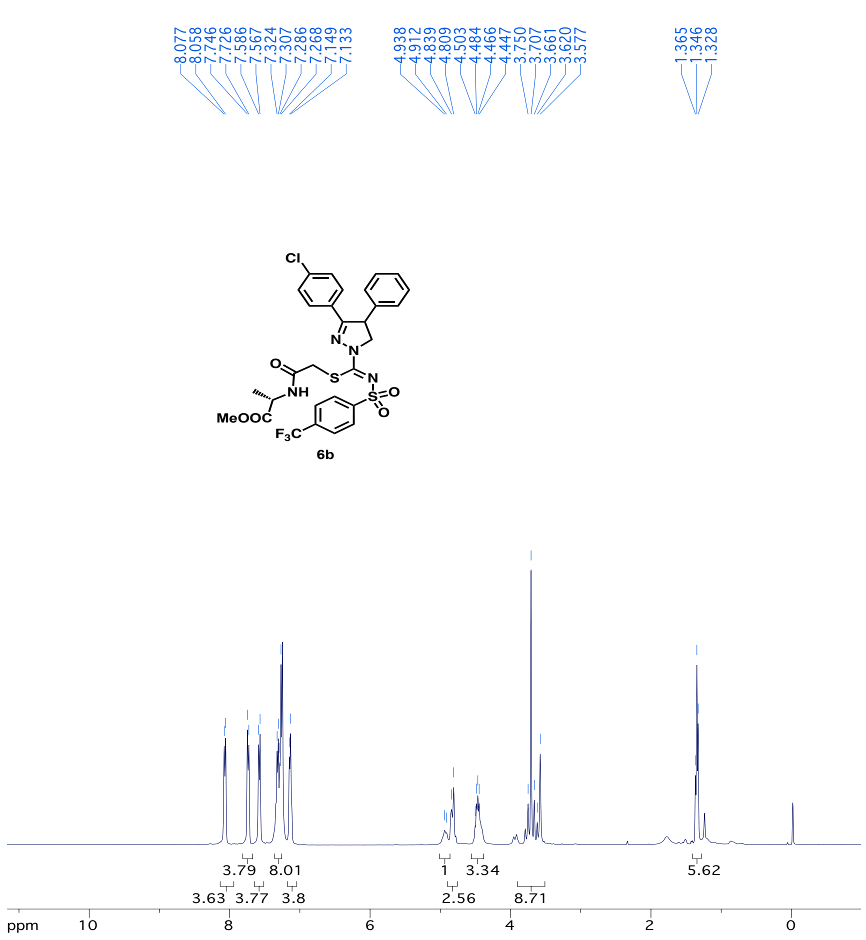

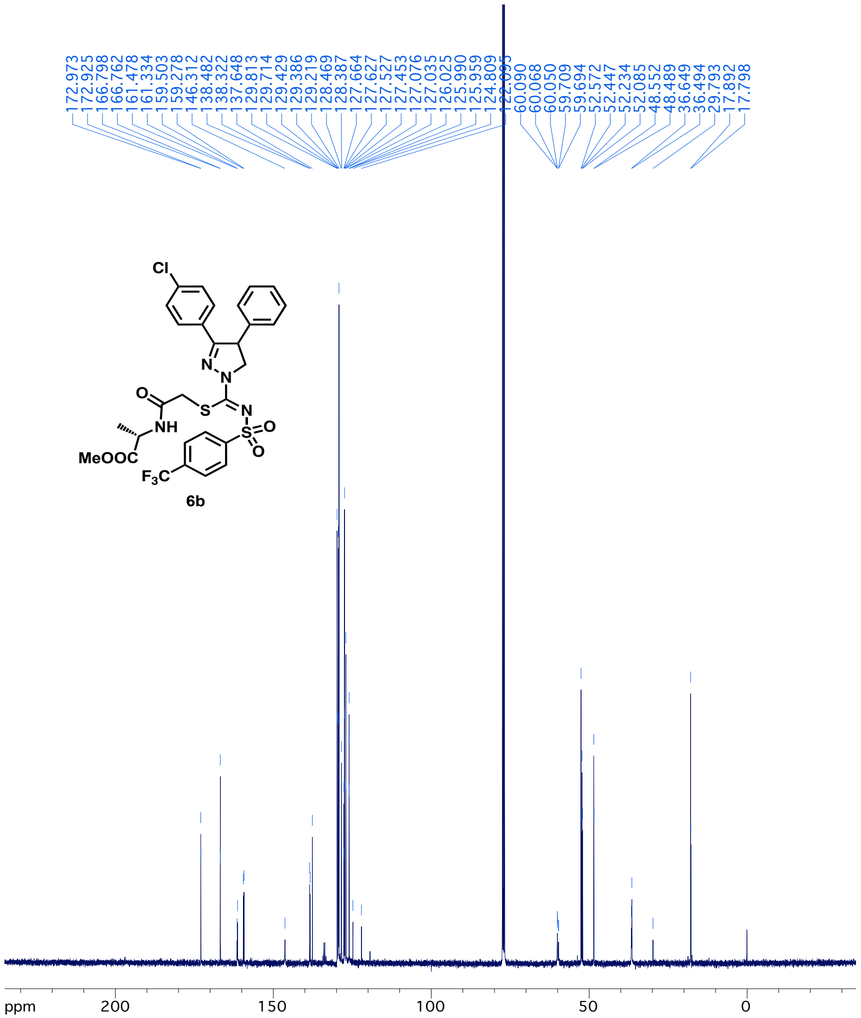

^{1}H-NMR and ^{13}C-NMR of Compound **6c**

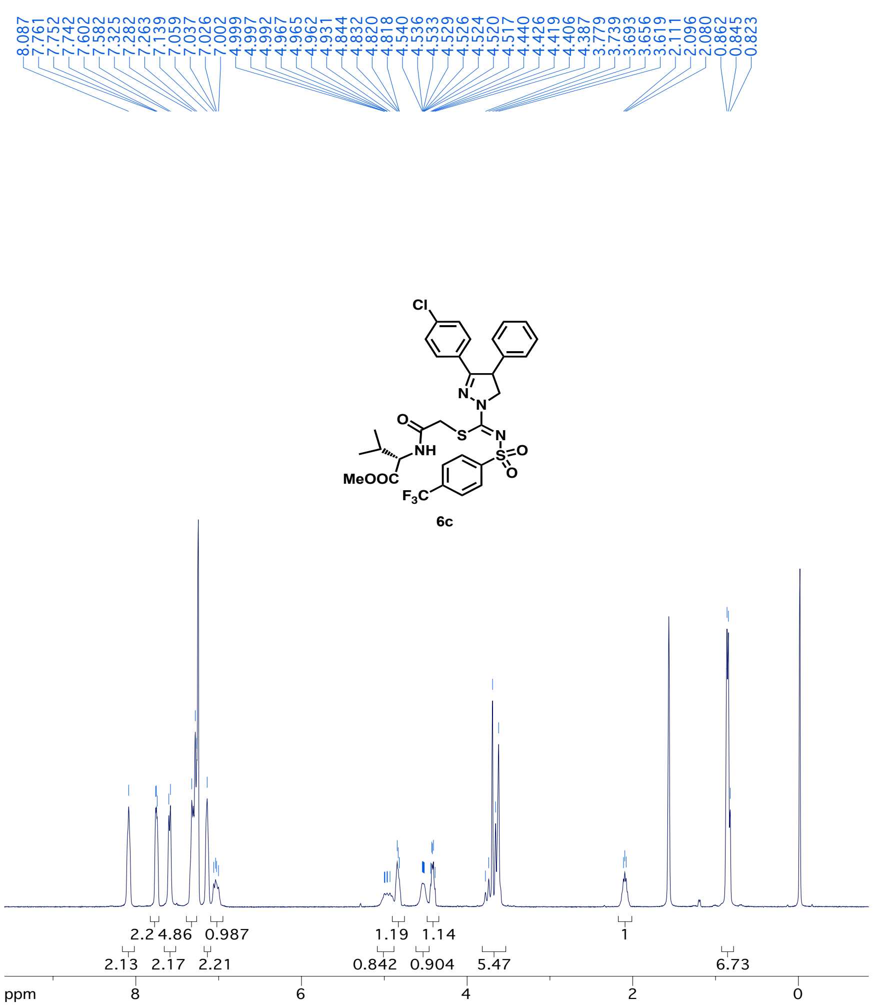

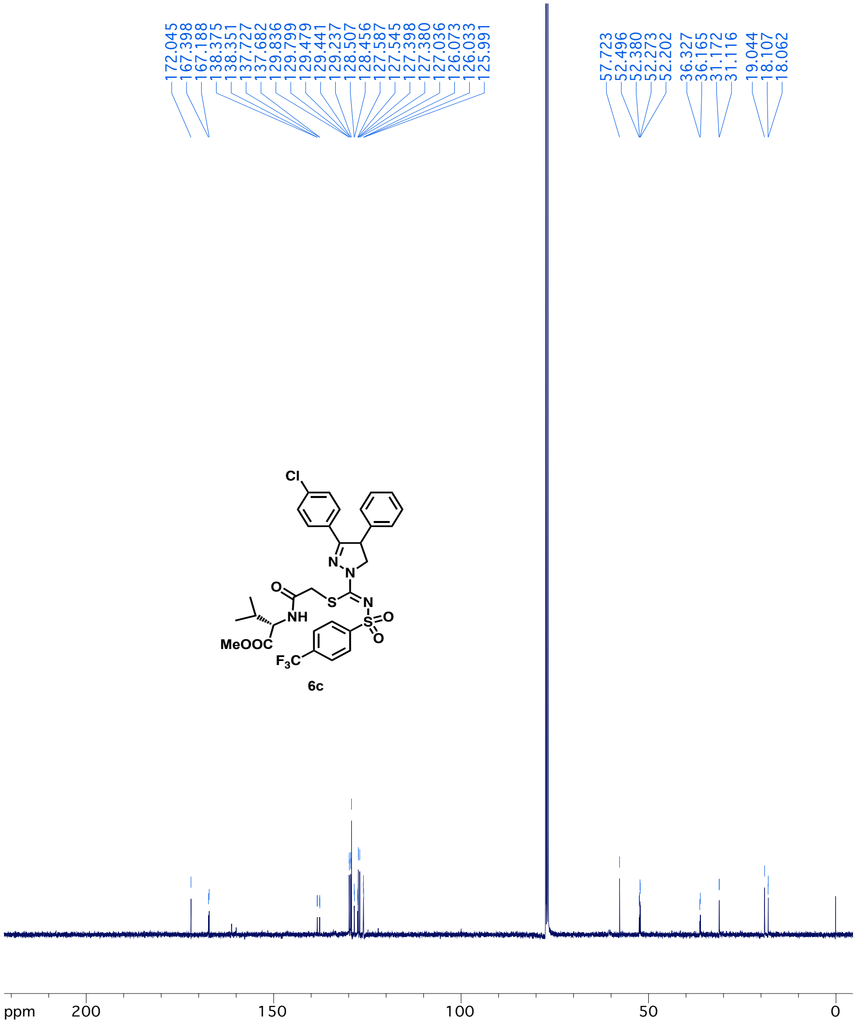

^{1}H-NMR and ^{13}C-NMR of Compound **6d**

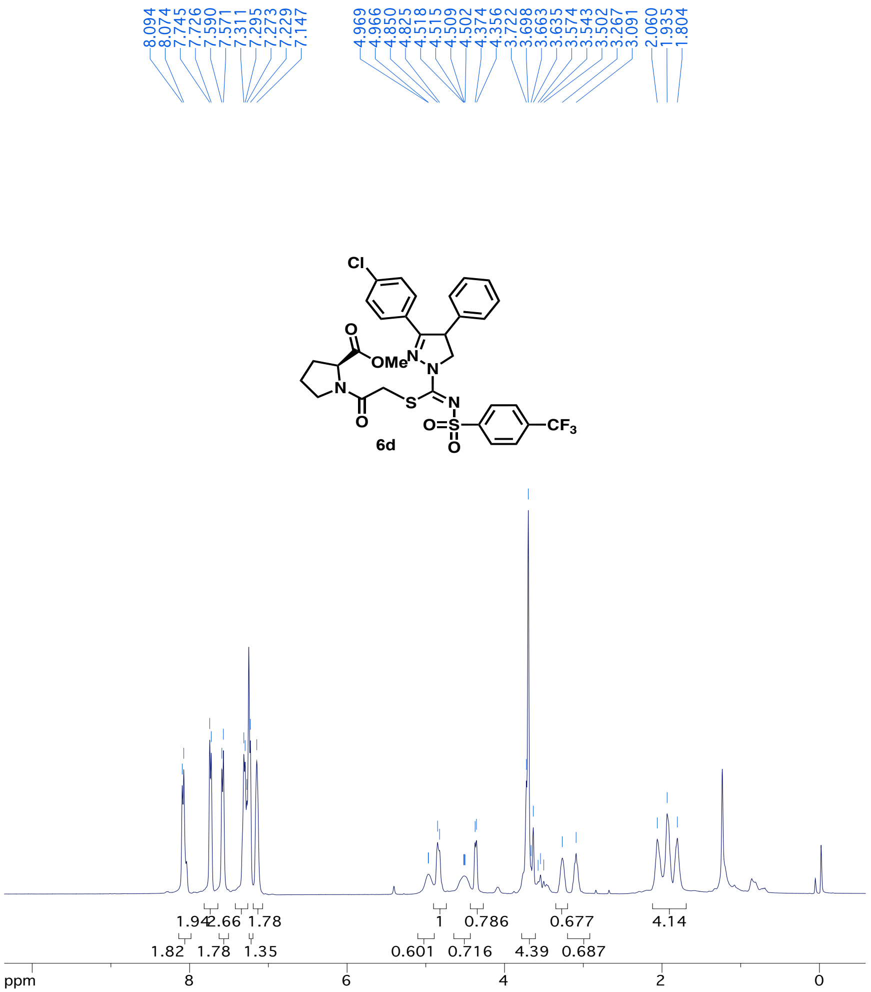

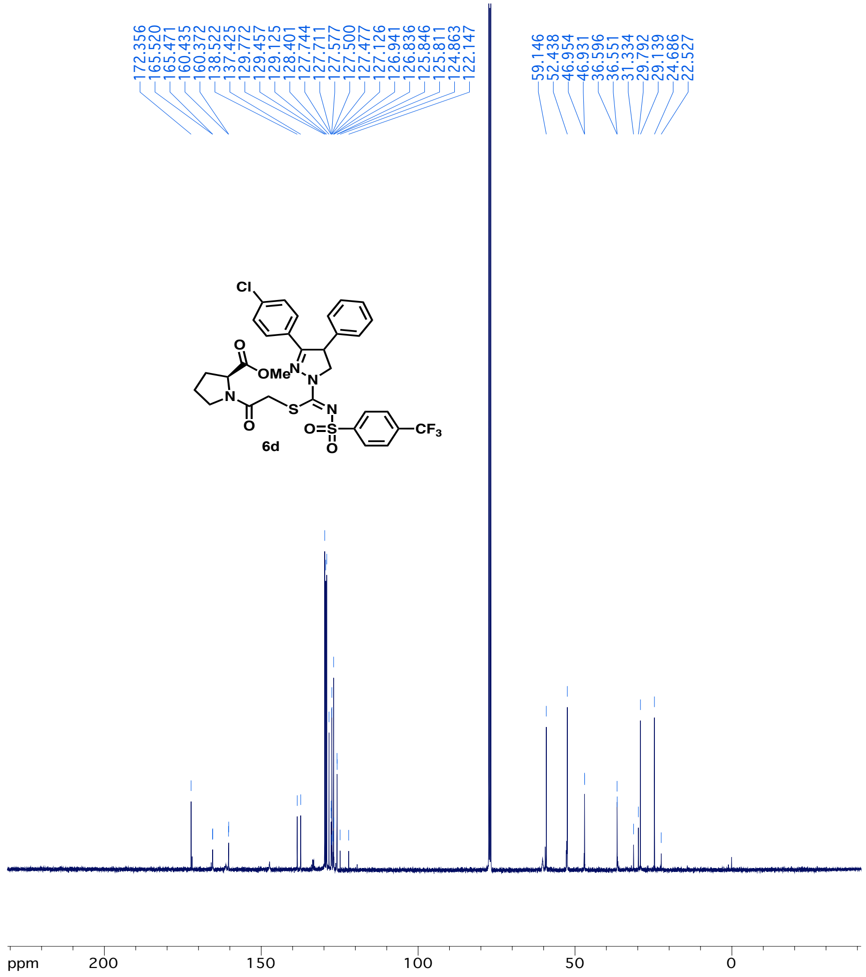

^{1}H-NMR and ^{13}C-NMR of Compound **6e**

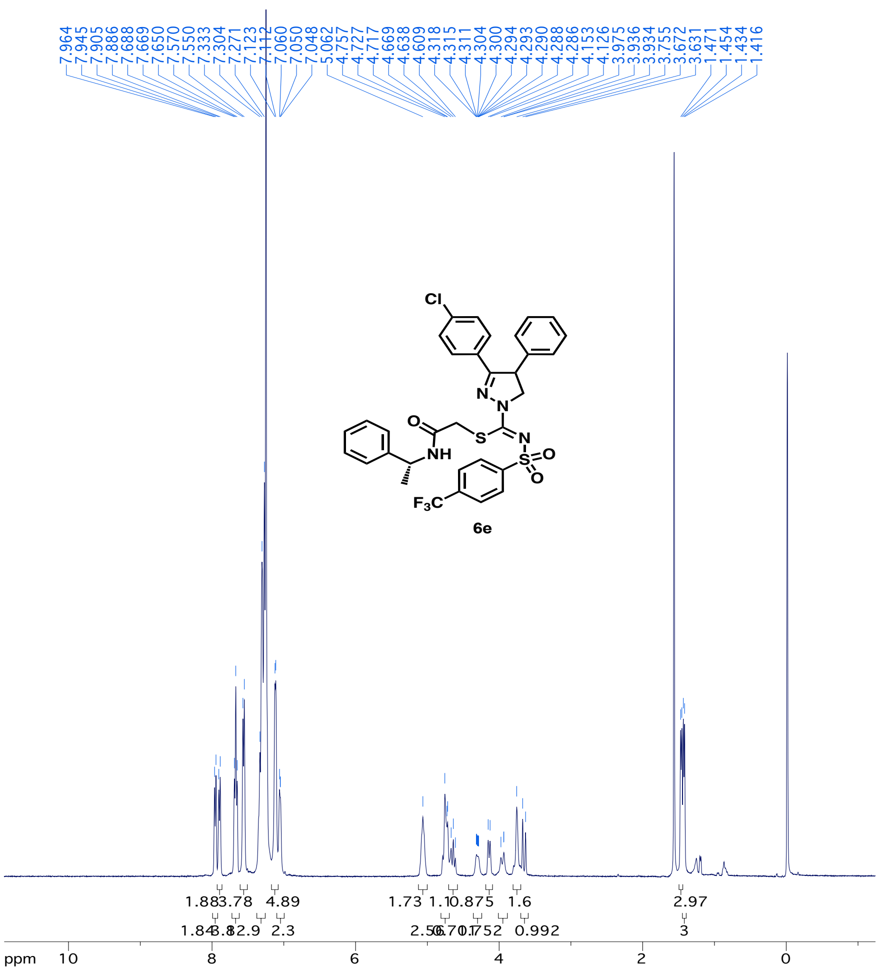

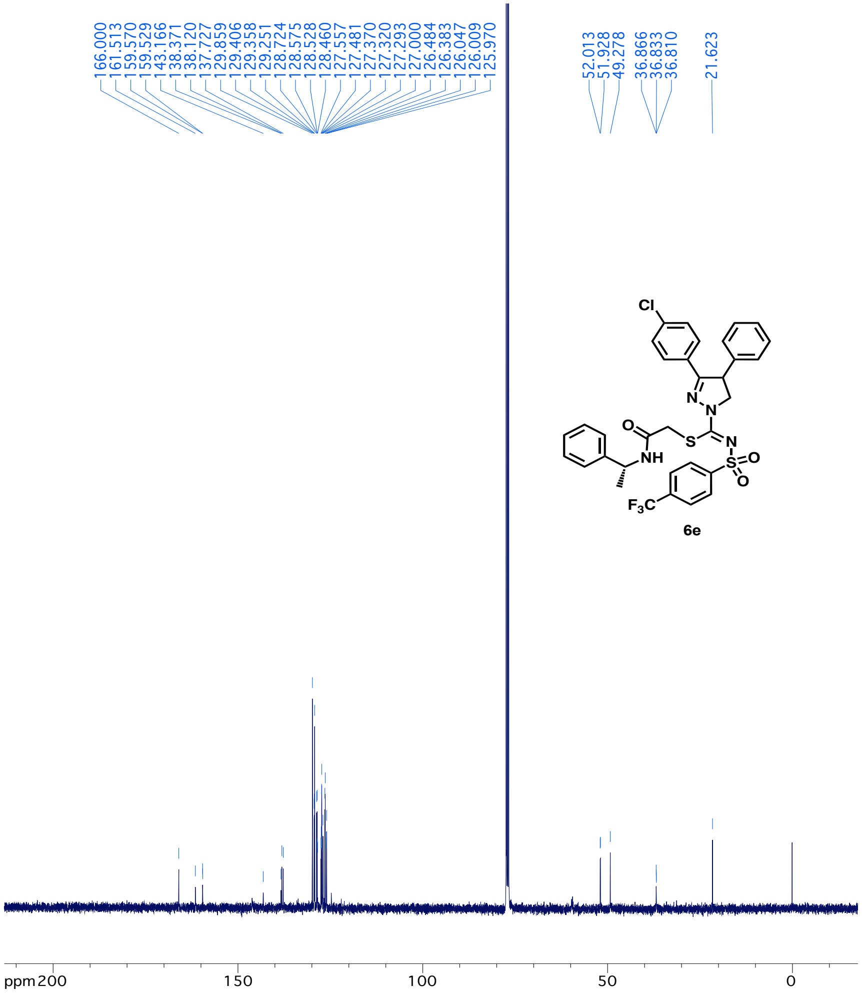

^{1}H-NMR and ^{13}C-NMR of Compound **6f**

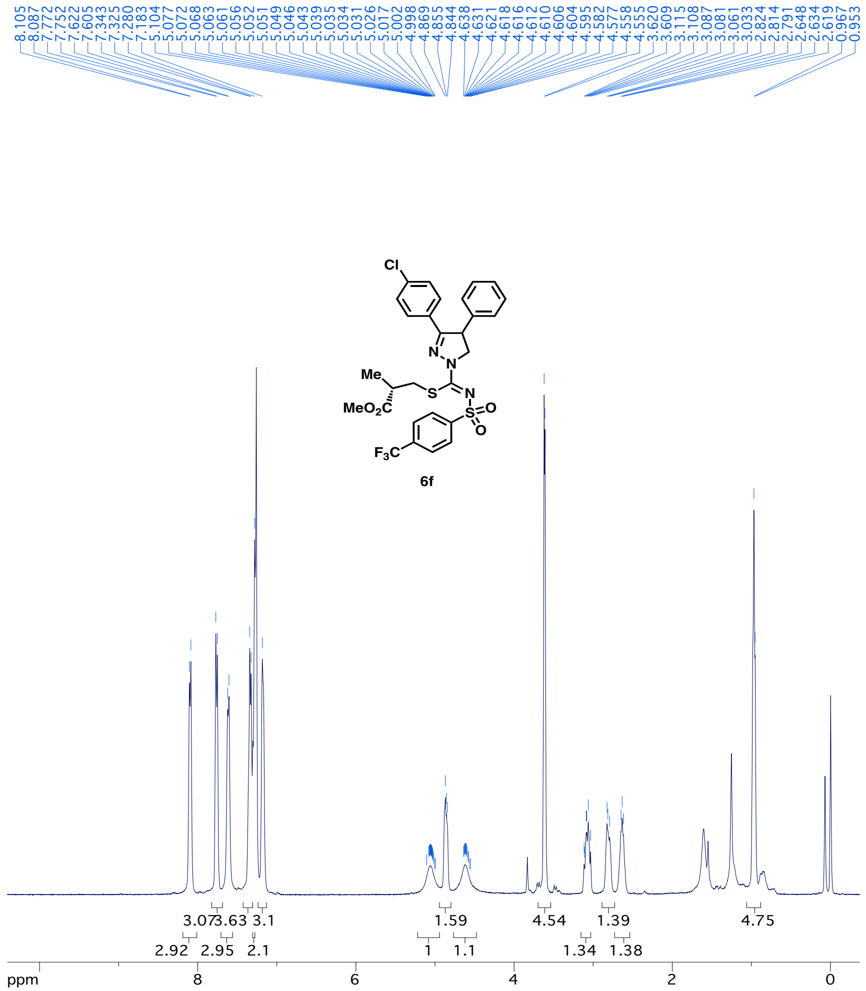

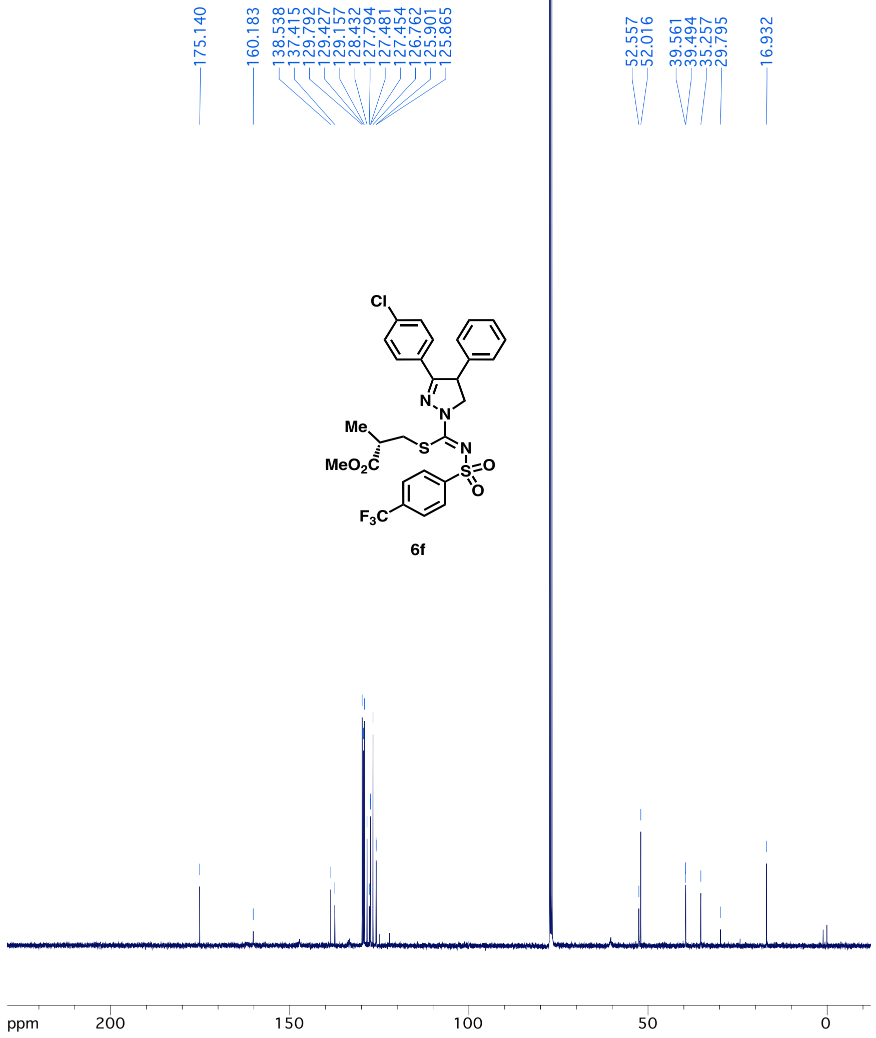

^{1}H-NMR and ^{13}C-NMR of Compound **7**

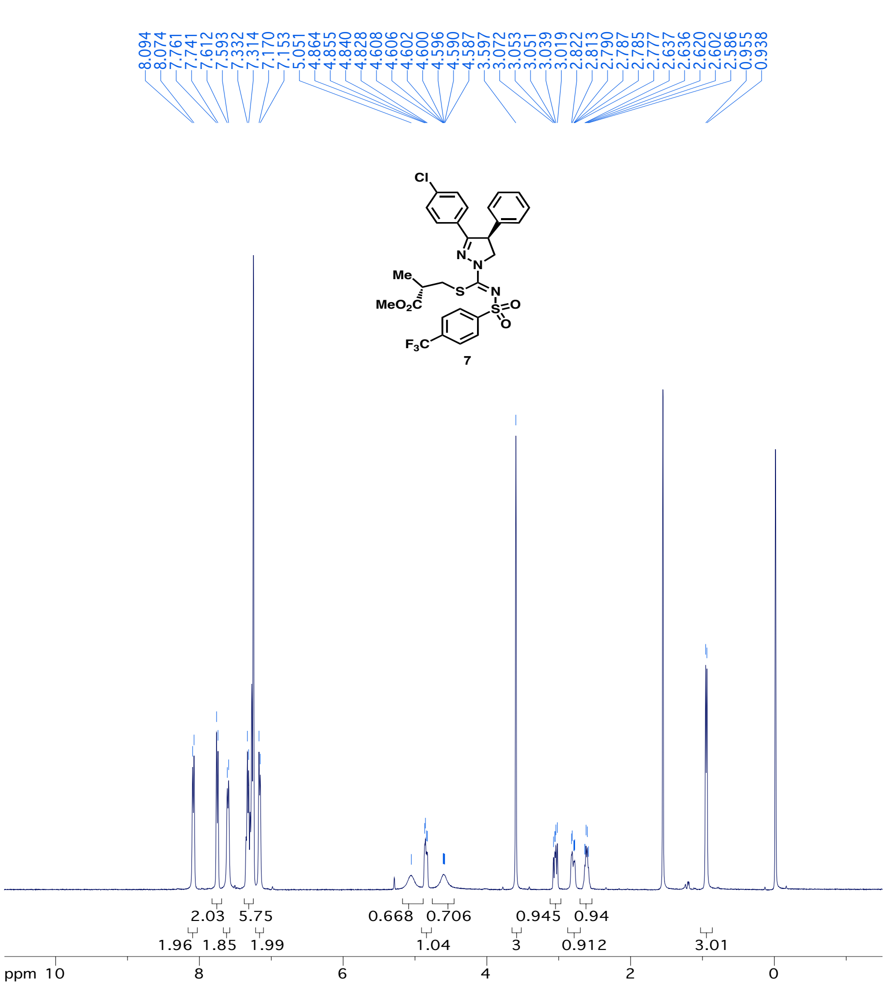

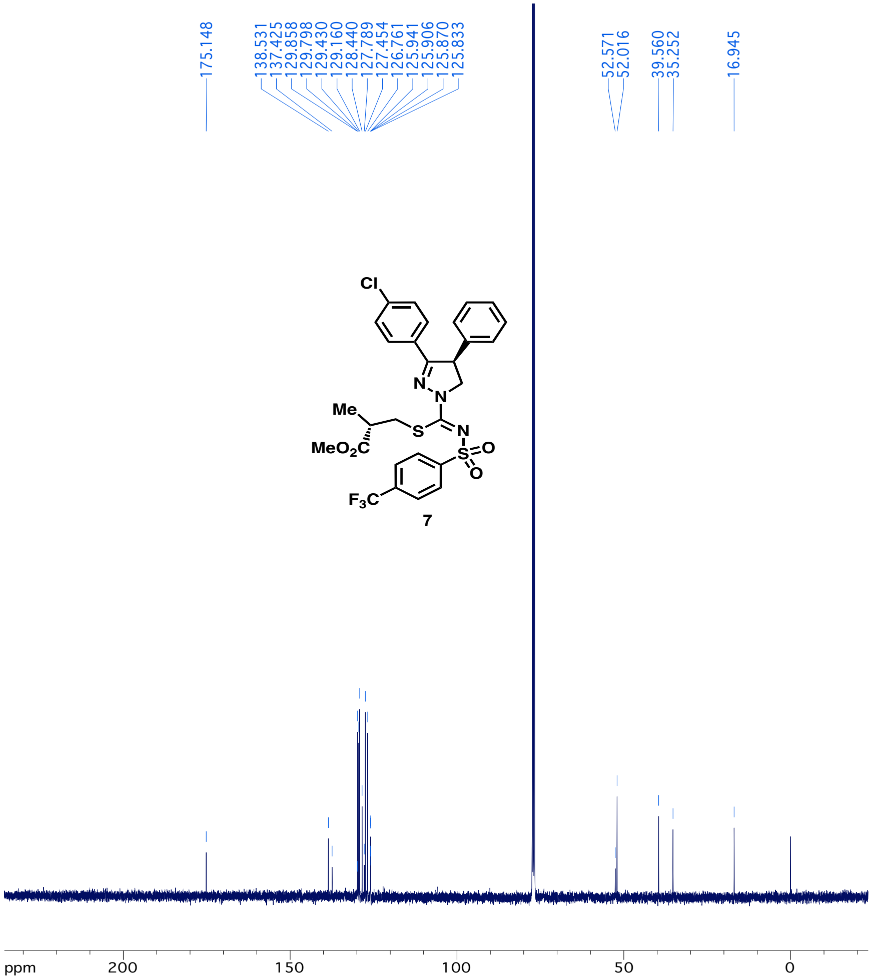

^{1}H-NMR and ^{13}C-NMR of Compound **8**

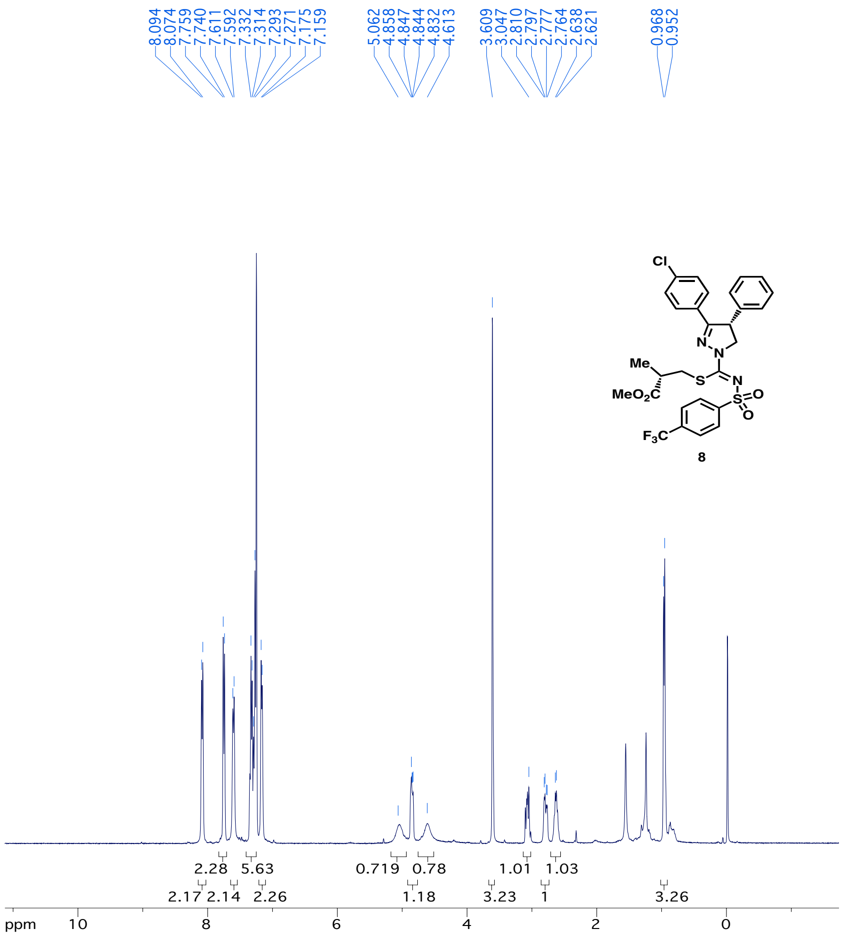

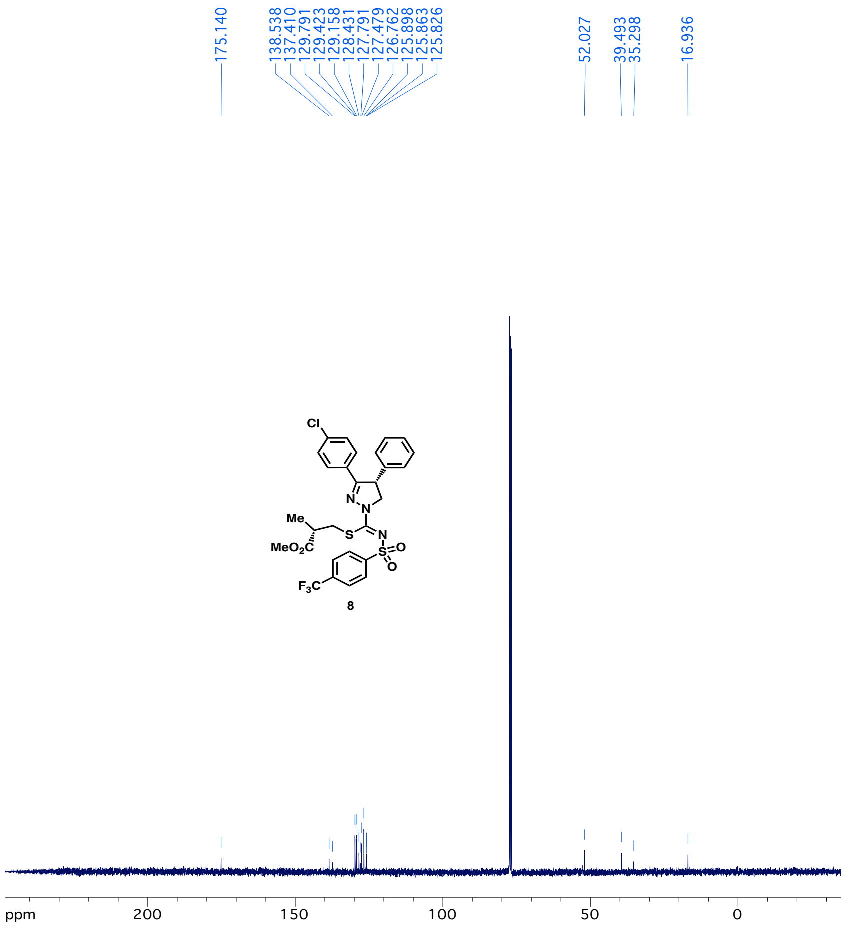

^{1}H-NMR for *S*-MRI-1867

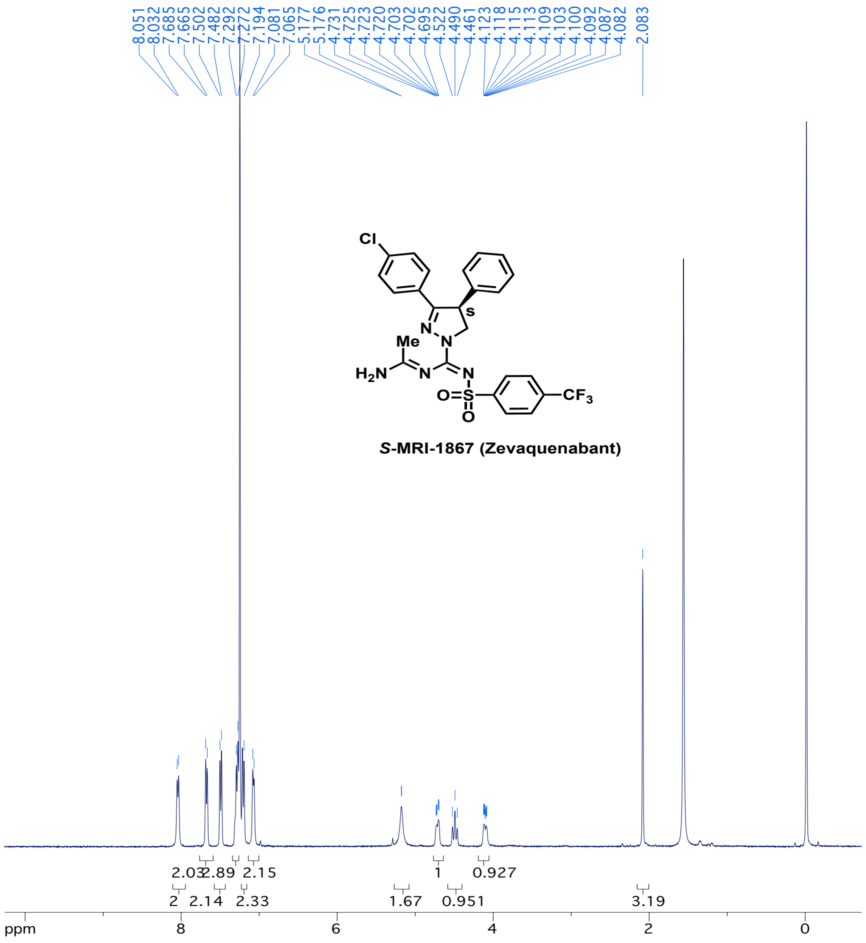

Chiral HPLC data for *S*-MRI-1867

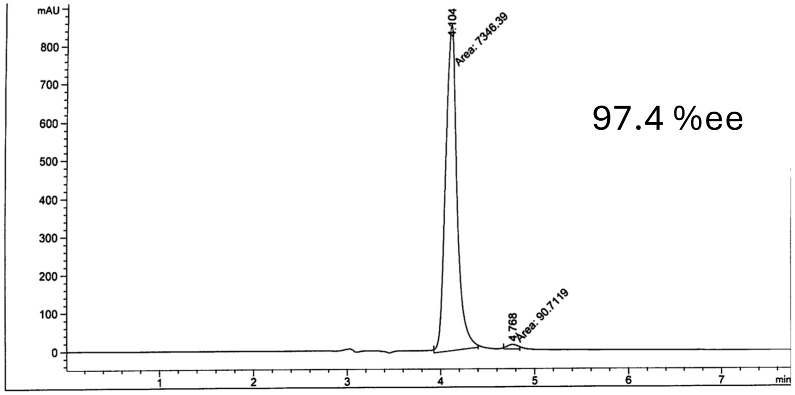

Area Percent Report

Sorted By : Signal
Multiplier : 1.0000
Dilution : 1.0000
Use Multiplier & Dilution Factor with ISTDs

Signal 1: DAD1 A, Sig=254,4 Ref=off

Peak #	RetTime [min]	Type	Width [min]	Area [mAU*s]	Height [mAU]	Area %
1	4.104	MM	0.1406	7346.39307	870.90625	98.7803
2	4.768	MM	0.1283	90.71194	11.78431	1.2197

Totals : 7437.10500 882.69056

Conditions: (*R*,*R*)-Whelk-O1 chiral column (250 mm x 4.6 mm/5μm): 100% EtOH 1.0 mL/min (254 nm)

Chiral HPLC data for *S*-MRI-1867

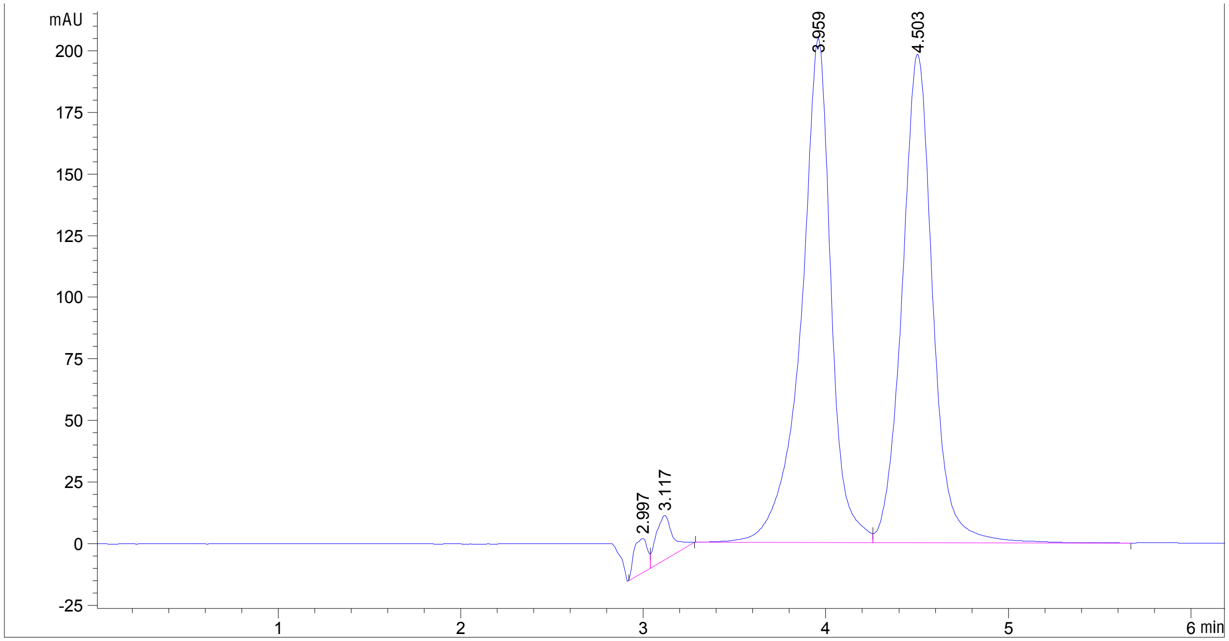

Conditions: (*R,R*)-Whelk-O1 chiral column (250 mm x 4.6 mm/5μm): 100% EtOH 1.0 mL/min (254 nm)

Chiral HPLC data for diastereomeric **6a** and **6b**

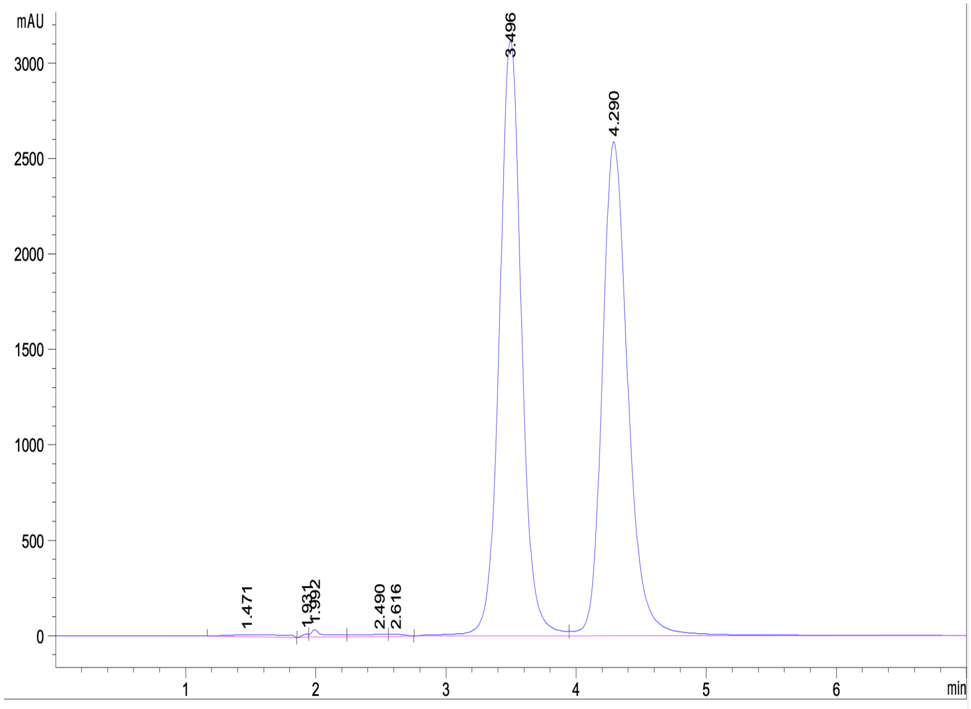

6a

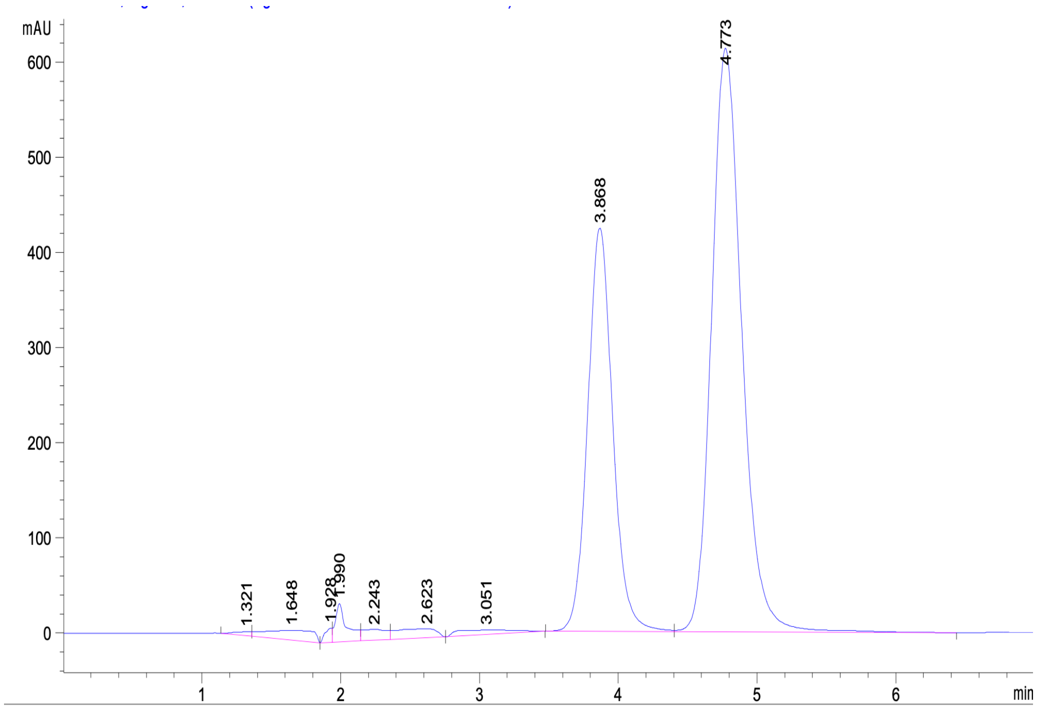

6b

Conditions: (*R*,*R*)-Whelk-O1 chiral column (250 mm x 4.6 mm/5μm): 100% EtOH 1.5 mL/min (254 nm)

Chiral HPLC data for diastereomeric **6c** and **6d**

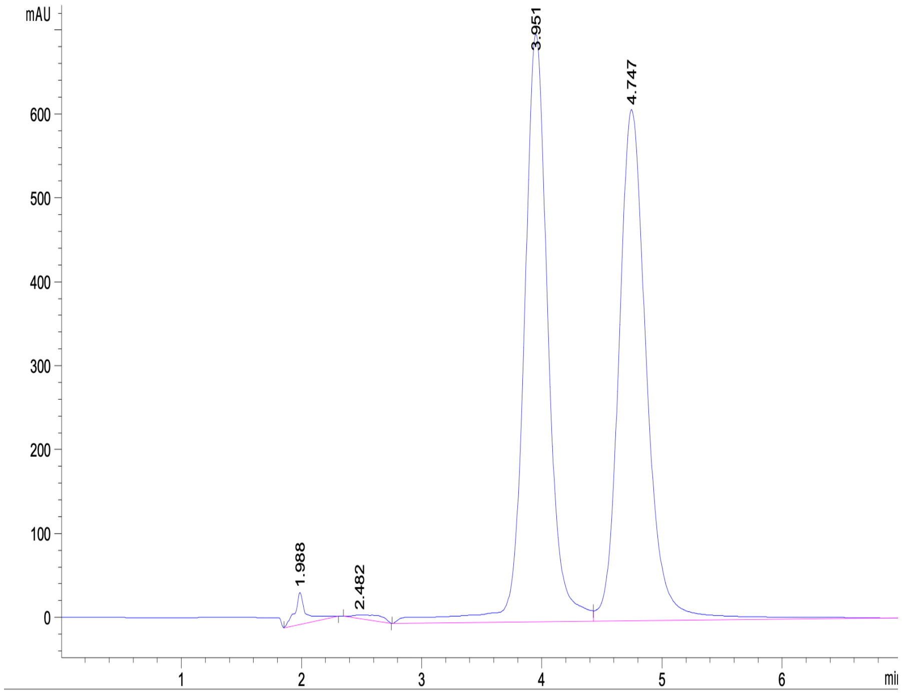

6c

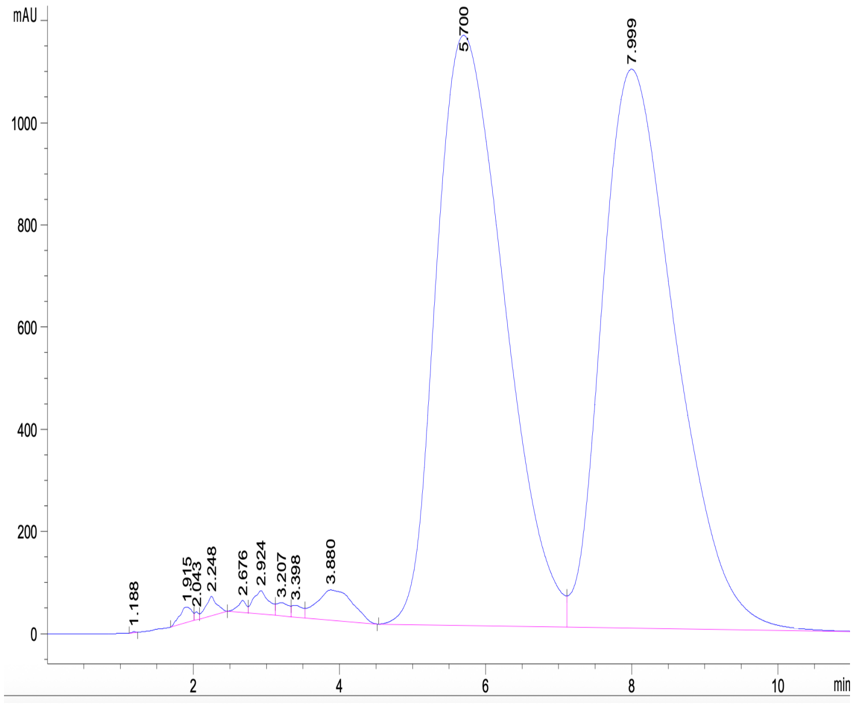

6d

Conditions: (*R*,*R*)-Whelk-O1 chiral column (250 mm x 4.6 mm/5μm): 100% EtOH 1.5 mL/min (254 nm)

Chiral HPLC data for diastereomeric **6e** and **6f**

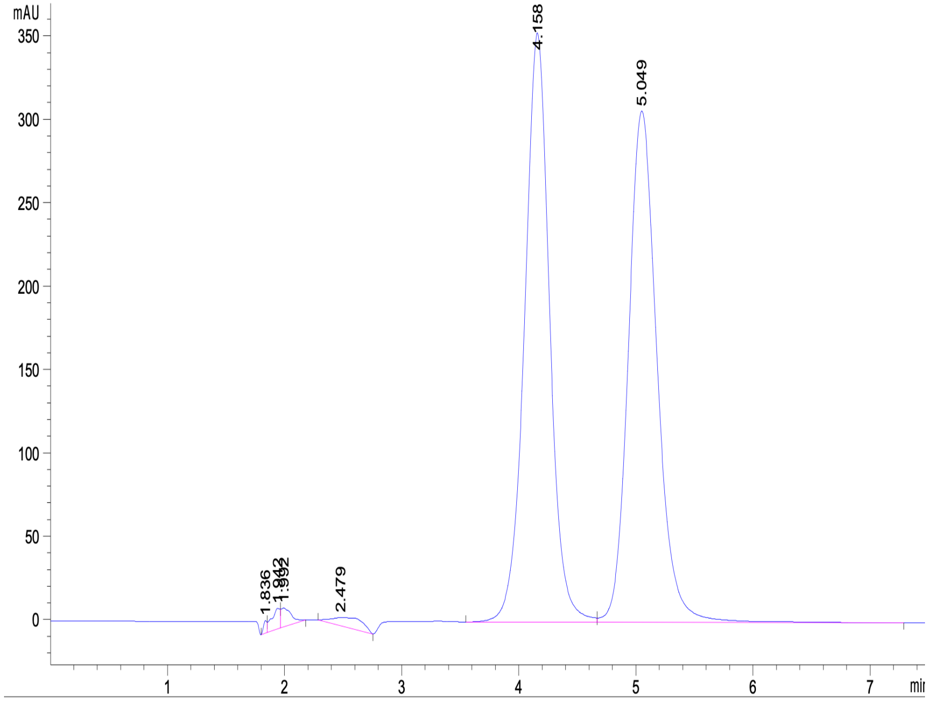

6e

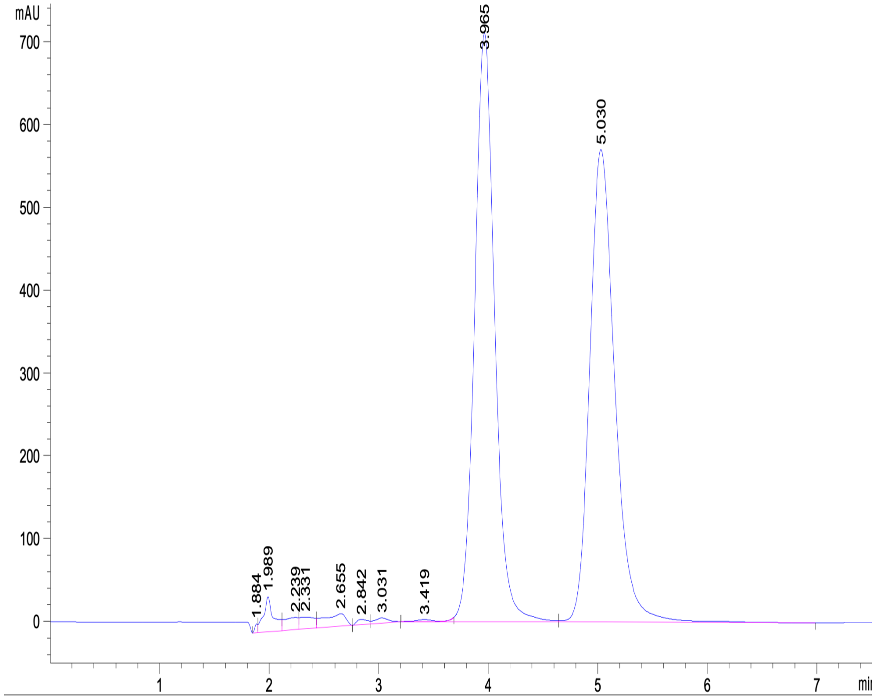

6f

Conditions: (*R*,*R*)-Whelk-O1 chiral column (250 mm x 4.6 mm/5µm): 100% EtOH 1.5 mL/min (254 nm)

Chiral HPLC data for diastereomeric **7 (S, S)**

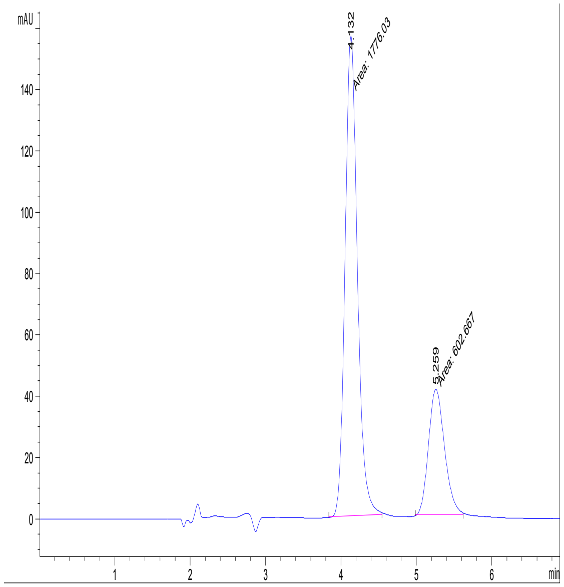

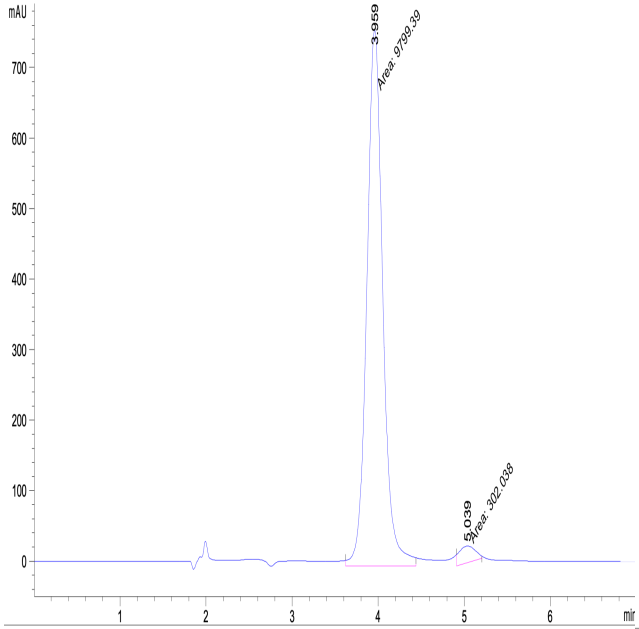

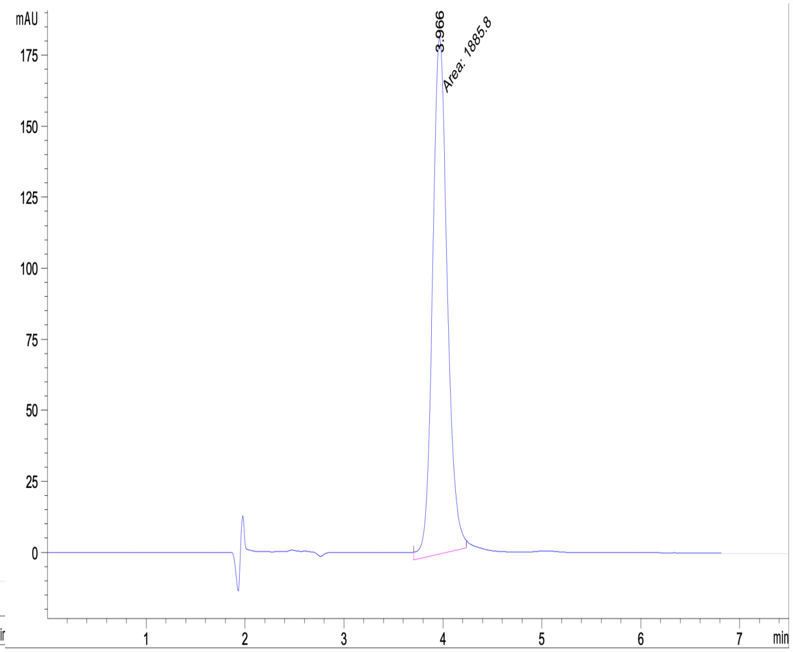

75:25 ratio S, S + S, R diastereomers

97:3 ratio S, S + S, R diastereomers

>99.9% S, S diastereomer

Conditions: (*R*,*R*)-Whelk-O1 chiral column (250 mm x 4.6 mm/5µm): 100% EtOH 1.5 mL/min (254 nm)

Chiral HPLC data for diastereomeric **8 (S, R)**

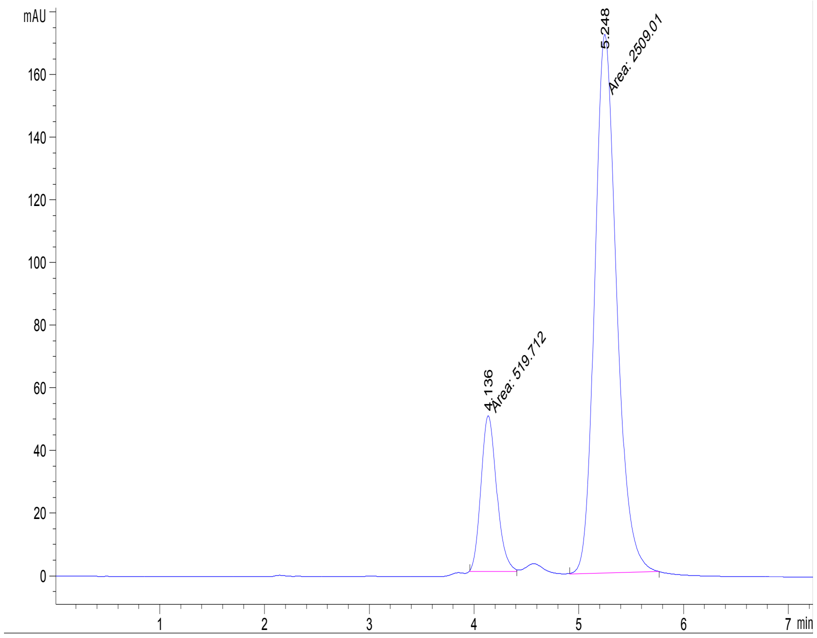

17:83 ratio S, S + S, R diastereomers

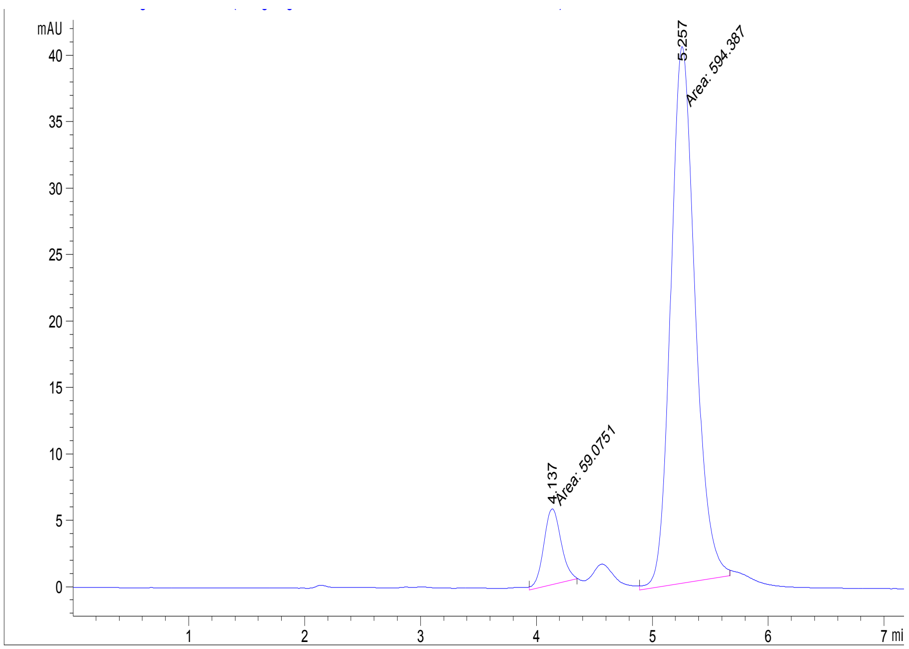

9:91 ratio S, S + S, R diastereomers

Conditions: (*R,R*)-Whelk-O1 chiral column (250 mm x 4.6 mm/5µm): 100% EtOH 1.5 mL/min (254 nm)
